# MicroRNA expression profile in bovine mammary gland parenchyma infected by coagulase-positive or coagulase-negative staphylococci

**DOI:** 10.1186/s13567-021-00912-2

**Published:** 2021-03-06

**Authors:** Emilia Bagnicka, Ewelina Kawecka-Grochocka, Klaudia Pawlina-Tyszko, Magdalena Zalewska, Aleksandra Kapusta, Ewa Kościuczuk, Sylwester Marczak, Tomasz Ząbek

**Affiliations:** 1 Department of Biotechnology and Nutrigenomics, Institute of Genetics and Animal Biotechnology Polish Academy of Sciences, ul Postepu 36A, 05-552 Jastrzębiec, Poland; 2 Department of Preclinical Sciences, Institute of Veterinary Medicine, Warsaw University of Life Sciences, ul Ciszewskiego 8, 02-786 Warsaw, Poland; 3 Department of Animal Molecular Biology, The National Research Institute of Animal Production, ul Krakowska 1., 32-083 Balice near Krakow, Poland; 4 Department of Applied Microbiology, Institute of Microbiology, Faculty of Biology, University of Warsaw, ul Miecznikowa 1, 02-096 Warsaw, Poland; 5 Experimental Farm, Institute of Genetics and Animal Biotechnology Polish Academy of Sciences, ul Postepu 36A, 05-552 Jastrzębiec, Poland

**Keywords:** MicroRNA, Target gene expression, Parenchyma, Mammary gland, Dairy cattle, Inflammation

## Abstract

MicroRNAs (miRNAs) are short, non-coding RNAs, 21–23 nucleotides in length which are known to regulate biological processes that greatly impact immune system activity. The aim of the study was to compare the miRNA expression in non-infected (H) mammary gland parenchyma samples with that of glands infected with coagulase-positive staphylococci (CoPS) or coagulase-negative staphylococci (CoNS) using next-generation sequencing. The miRNA profile of the parenchyma was found to change during mastitis, with its profile depending on the type of pathogen. Comparing the CoPS and H groups, 256 known and 260 potentially new miRNAs were identified, including 32 that were differentially expressed (*p* ≤ 0.05), of which 27 were upregulated and 5 downregulated. Comparing the CoNS and H groups, 242 known and 171 new unique miRNAs were identified: 10 were upregulated (*p* ≤ 0.05), and 2 downregulated (*p* ≤ 0.05). In addition, comparing CoPS with H and CoNS with H, 5 Kyoto Encyclopedia of Genes and Genomes pathways were identified; in both comparisons, differentially-expressed miRNAs were associated with the *bacterial invasion of epithelial cells* and *focal adhesion* pathways. Four gene ontology terms were identified in each comparison, with 2 being common to both *immune system* processes and *signal transduction*. Our results indicate that miRNAs, especially miR-99 and miR-182, play an essential role in the epigenetic regulation of a range of cellular processes, including immunological systems bacterial growth in dendritic cells and disease pathogenesis (miR-99), DNA repair and tumor progression (miR-182).

**Supplementary Information:**

The online version contains supplementary material available at 10.1186/s13567-021-00912-2.

## Introduction

Mastitis is one of the major health problems in dairy cattle herds worldwide, increasing costs and deteriorating animal welfare [1]. Its etiology can be bacterial, fungal or viral [2], with the main agents being staphylococci (Gram-positive) and *Escherichia coli* (Gram-negative) [3]. In turn, the staphylococci are divided into coagulase-positive (CoPS) and -negative (CoNS) species. CoPS bacteria, viz*.* some clinical isolates of *Staphylococcus aureus* and *S. intermedius*, thanks to producing of coagulases (prothrombin-activating factor) are able to cover themselves with fibrin upon contact with blood, protecting them from phagocytosis and other mechanisms of the immune system [4, 5]. Thus, CoPS use their own distinctive mechanisms to provoke pro-coagulant and pro-fibrinolytic reactions to avoid an offensive by the host immune system, survive and disseminate in the host's organism. What is more, coagulase which is produced at the early bacteria growth cycle allows to evade the immune system and persist, whilst staphylokinase, released later during the fast proliferation phase, enables escaping bacteria from the clot and causing systemic infection. Simultaneously, coagulase-negative bacteria characterized by a reduced persistence and dissemination rate. Thus, CoPS are able to use the host’s immune system to survive and multiply in the organism [4]. Staphylococcal enterotoxins are also one of the virulence factors [5].

The bacteria causing mastitis are divided into major pathogens, such as *S. aureus*, a contagious bacterium responsible for both clinical and chronic mastitis [5, 6], and minor pathogens, including various CoNS species which do not usually cause severe mastitis such as *Stahylococcus chromogenes*, *S. epidermidis*, *S. simulans*, *S. haemolyticus*, *S. sciuri*, and *S. xylosus* [7]. Waller et al. [8] found *S. chromogenes*, *S. epidermidis*, *S. simulans* and *S. haemolyticus* to be the most common etiological agents of mastitis. In addition, they report that *S. epidermidis*, *S. saprophyticus*, *S. chromogenes*, *S. simulans*, and *S. haemolyticus* tended to cause subclinical infection, while *S. hyicus* caused clinical mastitis. Contrary to Oviedo-Boyso [5] and Boulanger [6], Waller et al. [8] found *S. aureus* and *S. lentus* (CoNS bacteria) to exist only in clinical form, *S. arlettae*, *S. gallinarum*, *S. pseudintermedius* and *S. saprophyticus* caused only subclinical infection, while *S. hyicus*, *and S. warneri/pasteuri* caused both clinical and subclinical mastitis.

Pathogen invasion triggers both systemic and organ-specific immunity and affects the expression of a vast number of genes. The onset of inflammation, i.e. the innate response, is characterized by a predominance of macrophages, neutrophils, and natural killer cells. Leukocytes migrate to the mammary gland in response to cytokine signals; of these, tumor necrosis factor α (TNF-α) and interleukin 1β (IL-1β) have the greatest influence. If the innate response fails, udder tissues can also eliminate pathogens using a combination of innate and adaptive (specific) immunity: the innate immunity stimulates the specific response, which in turn uses multiple defense mechanisms to improve the effectiveness of the innate immune response [9]. The strength of the host response depends on the type of pathogen, its virulence, infection stage, and the resistance of the host itself, i.e. its genetics. Therefore, miRNA expression can have a considerable influence on host immunity during mastitis, and a thorough understanding of their activity plays a key role in understanding the etiology of the disease [1].

MicroRNAs (miRNAs) are short non-coding RNA oligonucleotides, and are commonly detected in plants, invertebrates and vertebrates. They are typically 21–23 nucleotides (nt) in length, and are transcribed from both coding and non-coding regions of the genome [10, 11]. miRNAs regulate the expression of other genes inhibiting their mRNA translation or degradation, resulting in a reduction in protein synthesis [10], and are thus are able to modulate the functions of various immune cells, such as neutrophils, monocytes and T cells throughout, regulating the transcription of host cytokines and intracellular signaling pathways [12]. When a pathogen binds to a toll-like receptor (TLR), several downstream transduction pathways, such as nuclear factor kappa-light-chain-enhancer of activated B cells (NF-κB), mitogen-activated protein kinases (MAPKs), and members of the Interferon Regulatory Factors (IRFs) family are activated, probably through the adaptor proteins of the myeloid differentiation factor 88 (MyD88) family [13]. Of the toll-like receptors (TLRs), TLR2 plays a major role in identifying staphylococci, as it recognizes lipoteichoic acid (LTA), one of the cell wall components of Gram-positive bacteria. Moreover, *S. aureus* can also be recognized by peptide receptors that bind mannose, ficolins and complement system molecules, and this process is probably regulated by miRNAs [14].

Some miRNAs may play crucial roles during mastitis. Different levels of expression of some miRNA target genes, such as interleukin-8 (*IL-8*) and granulocyte–macrophage colony-stimulating factor (*GM-CSF*) were found in udder tissue of cows with mastitis [6]. In addition, Li et al. [15] found miR-31 and miR-205 to be downregulated, and miR-223 upregulated, in mammary epithelial tissue infected by *S. aureus* compared to healthy controls.

As the genetic background of mastitis remains unknown, there is a need to better understand the regulation of immune processes during mastitis [1], including the role of miRNAs. To investigate their role in the context of the immune response in the mammary gland, we compare the miRNA expression profiles of 3 groups of mammary gland parenchyma derived from cow udder quarters: one group infected with CoPS, another infected with CoNS and another group composed of uninfected quarters (H).

Although some studies have been conducted on miRNA profile in udder tissues, only few have focused on chronic mastitis of naturally infected animals. Long-term infection aggravates cell and tissue damages, resulting even in injuries or necrotic changes in the mammary gland [1]. Therefore, it is important to stress that, contrary to most available publications, the present study is based on samples from animals which were naturally infected and with chronic inflammation. In addition, very few reports have so far examined the action of miRNAs during inflammation (mastitis) caused by staphylococci.

Of the many techniques currently used to measure the expression of miRNA, Next Generation Sequencing (NGS) is considered one of the most developed and the most suitable for measuring miRNA expression and detecting completely new, unknown miRNAs [16].

The main aim of the study was to identify miRNAs potentially engaged in infection-related pathways during mastitis by comparing the changes taking place in the mammary gland miRNAome during infection with CoPS or CoNS with those in H. The levels of miRNA expression between the CoPS and CoNS groups were also compared.

## Materials and methods

### Animals and samples

The experiment was carried out on 40 Polish Holstein–Friesian breed dairy cows of the black-and-white variety; all were between their 1^st^ and 4^th^ lactation and were maintained at the Experimental Farm of the Institute of Genetics and Animal Biotechnology in Jastrzębiec, near Warsaw. The herd is kept mainly for commercial purposes and the cattle is under constant veterinary supervision. Animal housing conditions were the same as those presented by Kościuczuk et al. [17]. As the farm uses a herd management and milk recording system, information was available on the somatic cell count (SCC) and the number and duration of antibiotic therapies for each animal. The samples were taken from animals at least one month after their last therapy, during the last stage of lactation (approx. 280 days, SD = 25), in a certified slaughterhouse in accordance with the herd management rules. All cows demonstrated chronic inflammation caused by bacterial pathogens and had experienced several unsuccessful antibiotic therapies or reproduction problems. Any samples derived from cows with acute mastitis, i.e. the udder was red, swollen or painful to the touch, or pus was present in the milk, were excluded from the analysis. Immediately after slaughter, 1 cm × 1 cm × 5 cm samples of mammary gland parenchyma, mainly composed of secretory epithelial tissue, were collected from deep in the middle of the secretory part of each udder quarter. To remove the remaining milk and blood, the samples were washed in ice-cold phosphate-buffered saline at pH 7 (Sigma-Aldrich, Missouri, USA), then rapidly frozen in liquid nitrogen.

Samples of foremilk were taken manually two days before slaughter in an aseptic manner, and subjected to microbiological examination. The samples (100 µL) were spread on Mannitol Salt Agar and Columbia Agar (agar supplemented with 5% sheep blood) (bioMérieux, Craponne, France) and incubated at 37 °C for 24 to 48 h. The isolates were phenotypically evaluated for colony morphology. Any bacterial species were identified using an Analytical Profile Index (API) test (bioMérieux). Staphylococcal coagulase production was determined using a tube test with rabbit plasma. Additionally, the identification of *S. aureus* strains was confirmed using a Slidex Staph-Kit (bioMérieux).

From further analysis 6 samples were excluded: 1 in which more than one bacterial species was identified (i.e. *S. aureus* and *Escherichia coli*), 1 in which streptococci were identified, and 4 samples with *E. coli*. Each experimental group consisted only of samples taken from udder quarters infected by only a single bacterial species. Samples from healthy udder quarters adjacent to infected ones were not used: only samples collected from cows with all 4 healthy udder quarters were used as controls. *S. aureus* was identified in 35.8% of milk samples, and CoNS bacteria in 32.8%. Among CoNS, *S. epidermidis* predominated in the material, being found in almost 45% of samples, followed by *S. sciuri* (13%), *S. vitulinus* (12%), *S. xylosus* (12%), *S. chromogenes* (10%)*,* and *S. lentus* (8%).

Based on the microbiological analysis results, the samples were assigned to 3 groups. Finally, 36 of 160 tissue samples were chosen for miRNA analysis. The first group was formed from samples collected from cows infected with CoPS (*S. aureus* only) (*N* = 21 from 14 cows). The second group included samples from cows infected with CoNS (*N* = 9 from 8 cows). The third group consisted of samples collected from cows without pathogenic bacteria in milk (H, *N* = 6 from 5 cows). More than one sample was selected from each cow to reduce the level of bias in the results.

### miRNAome libraries

Total RNA was extracted using the Direct-zol RNA MiniPrep kit (Zymo Research, Irvine, California, USA) according to the manufacturer’s protocol. RNA concentration and quality were measured using a NanoDrop 2000 spectrophotometer (Thermo Fisher Scientific, Waltham, Massachusetts, USA) and a 2200 TapeStation instrument (Agilent, Santa Clara, USA); only samples with ratio A260nm/230 nm between 1.8 and 2.2 and RNA integrity number (RIN) values greater than 7.5 were used. Following this, miRNA libraries were prepared from 950 ng of total RNA using a NEBNext Multiplex Small RNA Library Prep Set from Illumina (New England Biolabs, Massachusetts, USA), using the standard protocol. Briefly, 3′ adaptor ligation was followed by hybridization with the Reverse Transcription Primer and ligation with the 5′ adaptor. The obtained ligation products were reverse transcribed and subjected to PCR amplification with 12 different indexed primers provided with the above-mentioned kit. Each of these 12 primers contains a 6 nt long unique sequence (“index”), which allows for barcoding of single library to minimizes the contamination of the sample. Their detailed sequences are presented in the kit protocol provided by the manufacturer. The PCR amplification products were size-selected (Novex 6% TBE PAGE gel, [Invitrogen, Carlsbad, California, USA]), then precipitated and purified with ethanol (POCH, Gliwice, Poland). The final libraries were subjected to quantity and quality controls with a Qubit 2.0 Fluorometer (Thermo Fisher Scientific, Waltham, Massachusetts, USA) and a 2200 TapeStation instrument (Agilent, Santa Clara, California, USA), respectively. The libraries were stored at a 10 nM concentration at −20 °C until further use.

### miRNAome next-generation sequencing

The libraries of the 2 nM miRNAome and the PhiX control (libraries adjusted to 2 nM concentration) were denatured with 0.1 N NaOH, then diluted to 10.5 pM with pre-chilled HT1 buffer (hybridization buffer supplied by Illumina and New England Biolabs) on the sequencing day. The PhiX control library and the sample libraries were mixed and clustered on the Illumina Flowcell_v3 in a cBot cluster station (Illumina, San Diego, USA). Each library was sequenced on a HiScan SQ (36 cycles), according to the manufacturer’s protocol in two technical replicates. All of the above tests were delivered by Illumina.

### Data analysis

Parity was not found to have any influence on miRNA gene expression levels (preliminary analysis of variance), therefore this effect was not included in the final statistical model. FastQ converted read files were demultiplexed (bcl2fastq software, Illumina and New England Biolabs), and quality controlled using FastQC software [18]. The reads were then subjected to further processing and final miRNA identification using UEA sRNA Workbench V3.2, along with the embedded miRCat tool [19]. The detailed pipeline and applied parameters have been described previously [20]. Alignments were carried out with reference to the *Bos taurus* genome [21] and miRBase v21.0 [22, 23], whereas potentially novel miRNAs were additionally checked in the RNAcentral database [24] to exclude those belonging to other non-coding RNA species. Moreover, the identification of miRNA length and sequence variants (isomiRs) was performed by applying the isomiR-SEA software [25] with the default settings. The detected miRNAs, along with their isomiR sequences, were subjected to differential expression analysis using DESeq2 software [26], according to the producer’s instructions. The identity numbers of individual cows were included in the analysis so that the presence of two samples from the same animal was considered in the DESeq2 analysis.

The numbers of miRNA genes shared between groups were calculated using Venn diagram drawing software [27].

### Analysis of miRNA over-represented biological pathways

The differentially-expressed (DE) miRNAs (*p* ≤ 0.05) found in the previous step of the analysis were subjected to further analysis. Their target genes and biological processes involved were identified using the mirPath v.3 DIANA Tools web application [28]. DIANA—TarBase v.7.0 [29], with its experimentally validated target genes, was used as the reference database for the targeted genes. KEGG (Kyoto Encyclopedia of Genes and Genomes) pathway and GO (Gene Ontology) analyses were carried out with miRBase (21.0) [30] human homologs because of the lack of data for cattle miRNAs. To additionally visualize the interaction networks of the identified DE miRNAs and their target genes, the miRNet network-based tool was used [31].

## Results

### Characterization of the miRNA expression profile of the udder tissue

Between 2.9 M and 6.7 M sequence reads were obtained and filtered in individual samples. For the comparison of the CoPS and H groups, 2.2 M and 4.5 M sequences (mean 74%) were mapped to the reference *Bos taurus* genome. Further analysis identified 256 distinct known miRNAs deposited in miRbase, and 260 unique, potentially novel miRNAs (Additional file 1). Among the identified miRNAs, 33 sequences were localized on the strand opposite to that deposited in miRBase (referred as −3p and −5p). For the comparison of the CoNS and H groups, between 2.2 M and 4.4 M sequences (mean 78%) were mapped to the reference genome. Further analysis allowed the identification of 242 distinct known miRNAs, including 29 miRNAs that come from the opposite arm of a pre-miRNA hairpin (identified as miRNAs*), and 171 unique, potentially novel miRNAs (Additional file 2). For both comparisons, the most commonly-observed miRNA length was 22 nts, followed by 21 and 23 nts.

### Alterations of the miRNAome during CoPS and CoNS infection of mammary gland parenchyma

**Figure 1:**
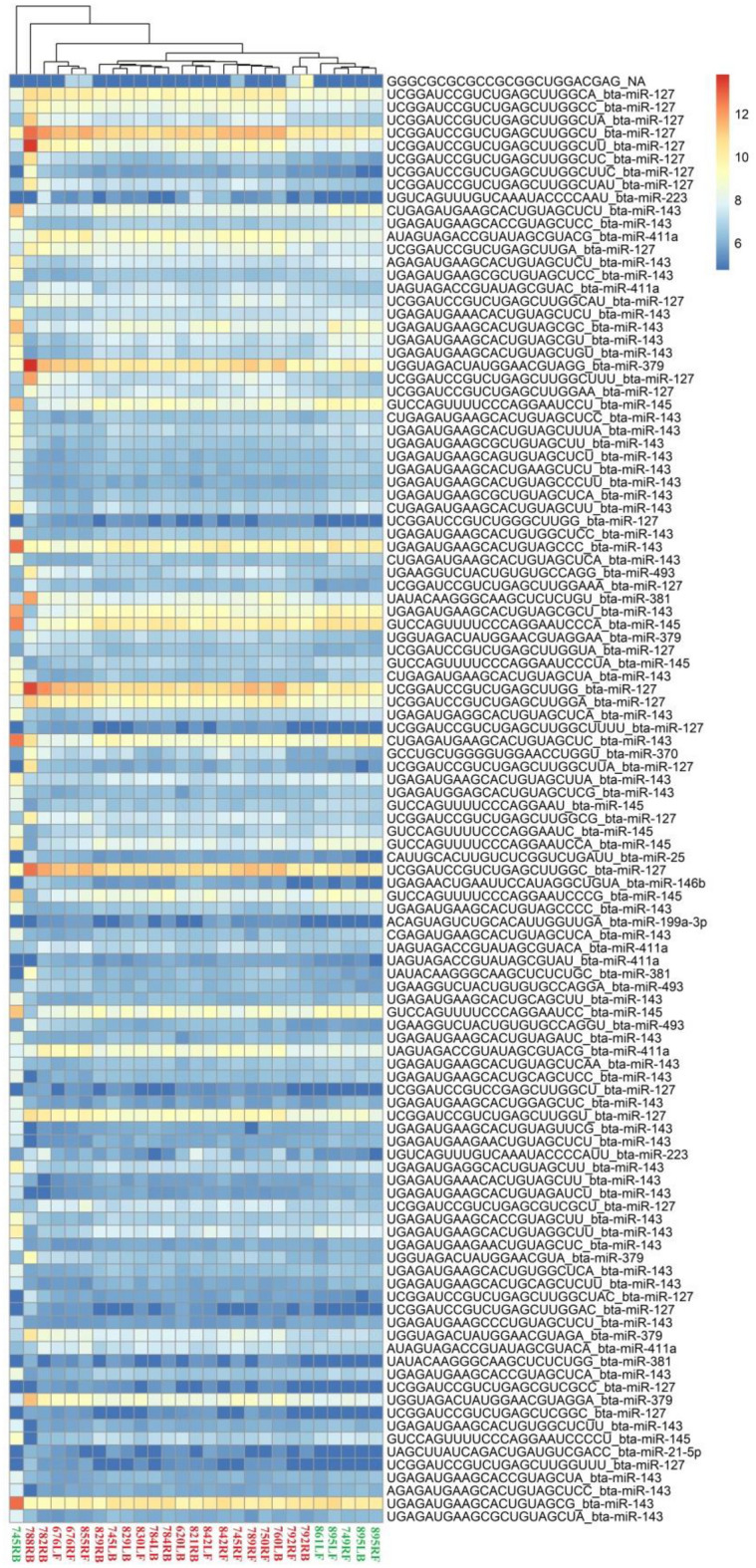
**The expression pattern of the most significant miRNAs and isomiRs in coagulase-positive staphylococci udder infection.** R Package pheatmap [66]. CoPS—coagulase-positive staphylococci (red numbers), H—healthy group, without bacteria (green numbers). CoPS group: 620LB, 676LF, 676RF, 745LB, 745RF, 750RF, 760LB, 782RB, 784LB, 784RB, 788RB, 789RF, 792RF, 792RB, 821RB, 829LB, 829RB, 830LF, 842LF, 842RF, and 855RF. Healthy group: 861LF, 895LF, 895LB, 895RB, 745RB, and 749RF. RF—right front mammary gland quarter, RB—right back mammary gland quarter, LF—left front mammary gland quarter, LB—left back mammary gland quarter.

DESeq2 analysis of the udder parenchyma tissue infected with CoPS allowed the identification of 32 DE miRNAs (*p* ≤ 0.05), including 2 potentially new miRNAs, compared to H. Of these, 27 were upregulated, including 99 isomiRs, while 5 were downregulated, including 104 isomiRs. The number of DE isomiRs per miRNA ranged from one isomiR (e.g., bta-let-7c) to 79 (e.g., bta-miR-143). Moreover, all DE isomiRs stemming from a single miRNA demonstrated the same direction of expression change in the CoPS vs*.* H comparison. Detailed data on the differential expression analysis are shown in Additional file 3, while the most significant (*p* ≤ 0.01) DE miRNAs and their isomiR sequences are given in Figure 1.

**Figure 2:**
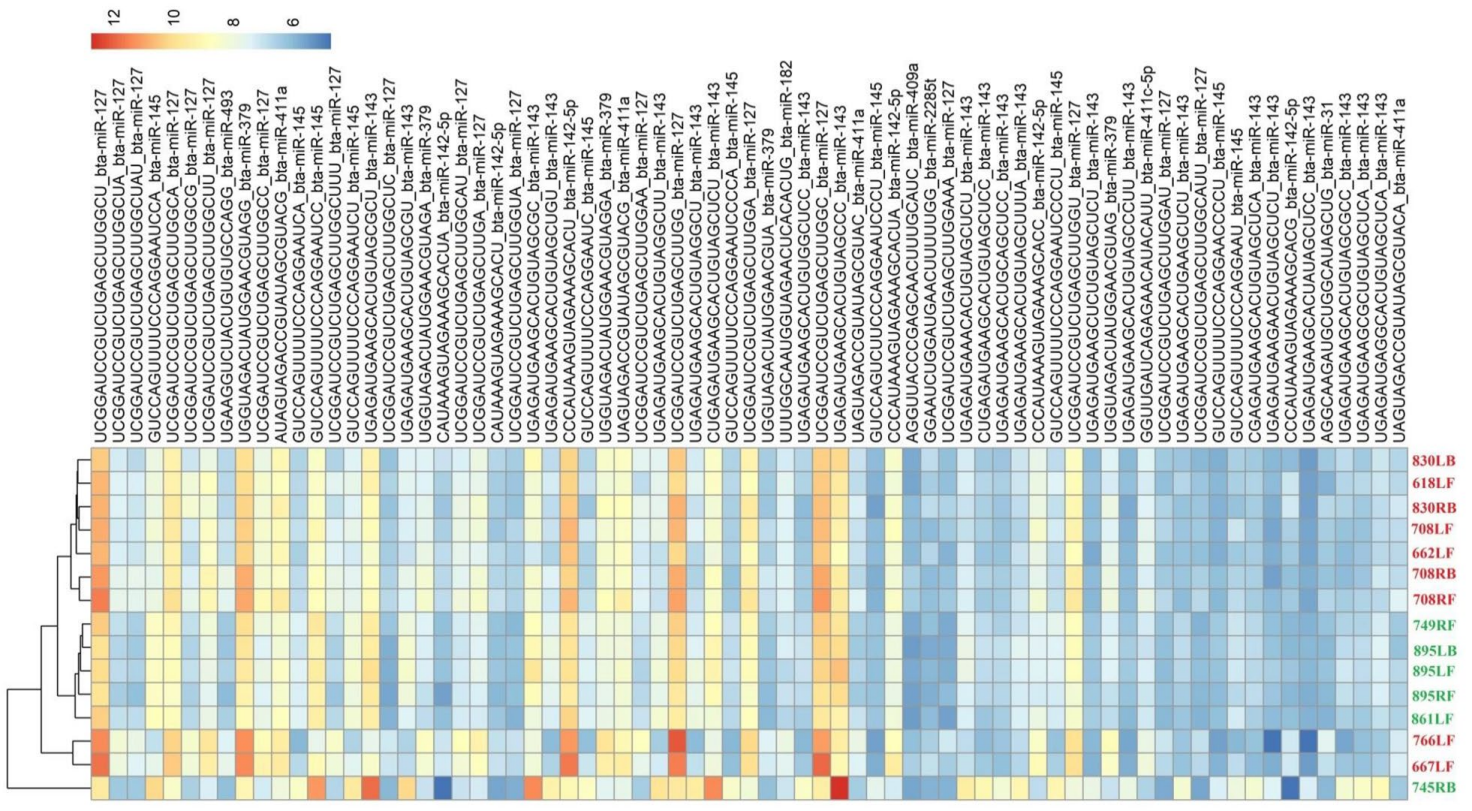
**The expression pattern of the most significant miRNAs and isomiRs in coagulase-negative staphylococci udder infection**. R Package pheatmap [66]. CoNS—coagulase-negative staphylococci (red numbers), H—healthy group, without bacteria (green numbers). CoNS group: 618LF, 662LF, 667LF, 708LF, 708RF, 708RB, 766LF, 830LB, and 830RB. Healthy group: 861LF, 895LB, 895RF, 745RB, and 749RF. RF—right front mammary gland quarter, RB—right back mammary gland quarter, LF—left front mammary gland quarter, LB—left back mammary gland quarter.

DESeq2 analysis of the udder parenchyma tissue infected with CoNS compared to the H group revealed 12 miRNAs that were DE (*p* ≤ 0.05) (bta-miR-127, bta-miR-142-5p, bta-miR-143, bta-miR-145, bta-miR-182, bta-miR-2285t, bta-miR-31, bta-miR-379, bta-miR-409a, bta-miR-411a, bta-miR-411c-5p, and bta-miR-493): of these, 10 were upregulated, consisting of 41 isomiRs, while 2 were downregulated, including 32 isomiRs. The number of DE isomiRs per miRNA ranged from one (e.g., bta-miR-2285t) to 22 (e.g., bta-miR-143). More detailed results of the analysis are presented in Additional file 4, while the most significant (*p* ≤ 0.05) DE miRNAs, together with their isomiR sequences, are shown in Figure 2.

**Figure 3:**
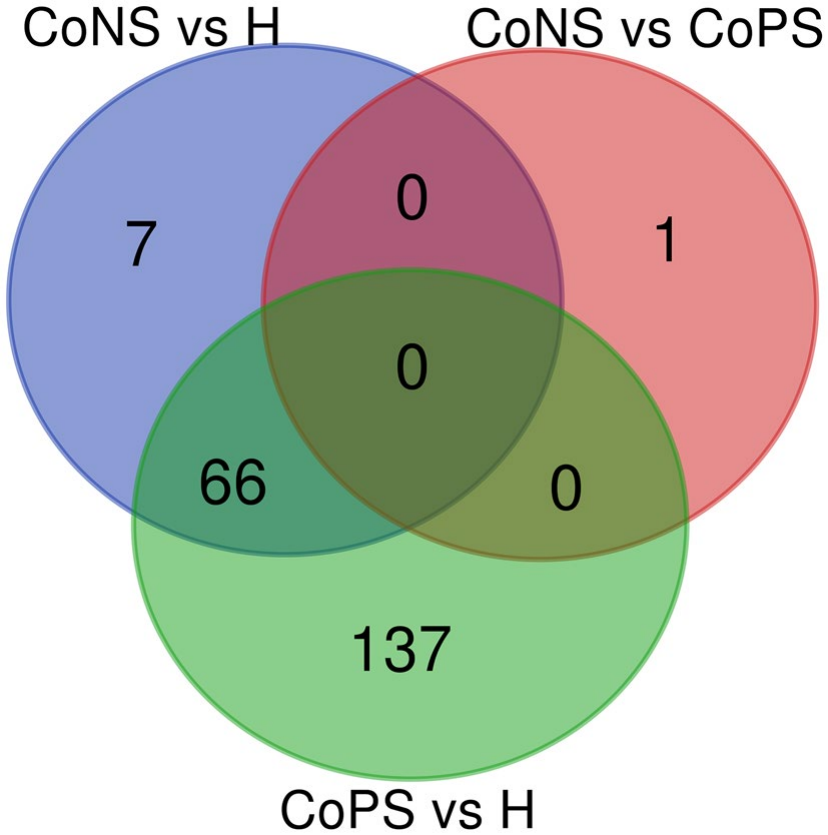
**The number of differentially expressed miRNAs in all comparisons.**

The analysis revealed 11 unique miRNAs and 66 isomiRNAs to be common between CoPS vs. H and CoNS vs. H. However, only one miRNA, bta-miR-106b-3p*, was DE in this comparison, being upregulated in CoPS (*p* ≤ 0.05). This is a passenger strand of the duplex marked by an asterisk. Interestingly, this miRNA was not DE in the other comparisons. All DE isomiRs originating from one miRNA demonstrated the same direction of expression changes in both comparisons (i.e. CoPS vs. H and CoNS vs*.* H). For all comparisons, the sequences and names of the unique and common miRNA genes are listed in Additional file 5 while the number of DE miRNAs are shown in Figure 3.

### Differentially-expressed miRNAs engaged in infection-related pathways in CoPS vs. H comparison

**Figure 4:**
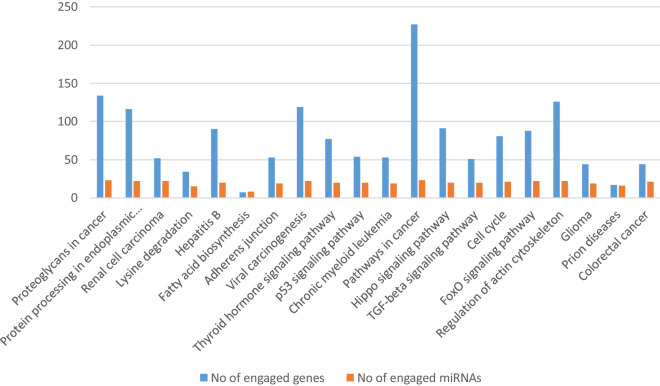
**The first 20 pathways according to their** ***p*-value for the CoPS vs. H comparison.**

**Figure 5:**
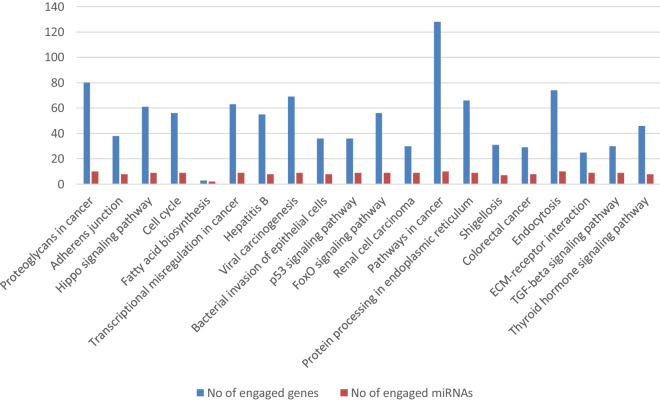
**The first 20 pathways according to their** ***p*-value for the CoNS vs. H comparison.**

The genes targeted by the identified DE miRNAs were then used for pathway and GO category analyses. A number of KEGG pathways were found to be over-represented in the CoPS vs*.* H (Additional file 6) and CoNS vs*.* H comparisons (Additional file 7), and various GO categories for the CoPS vs. H (Additional file 8) and CoNS vs. H (Additional file 9) comparisons. The first 20 pathways with the number of engaged genes and miRNAs are presented according to their p-value for CoPS vs. H in Figure 4 and for CoNS vs. H in Figure 5.

**Figure 6:**
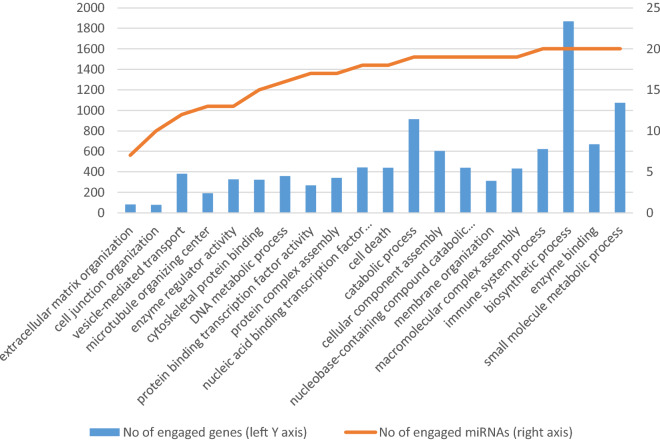
**The first 20 GO terms according to their *p*-value for the CoPS vs. H comparison.**

**Figure 7:**
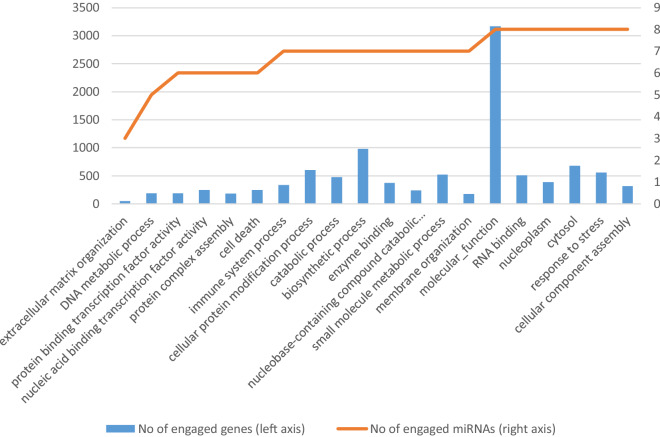
**The first 20 GO terms according to their** ***p*-value for the CoNS vs. H comparison.**

**Figure 8:**
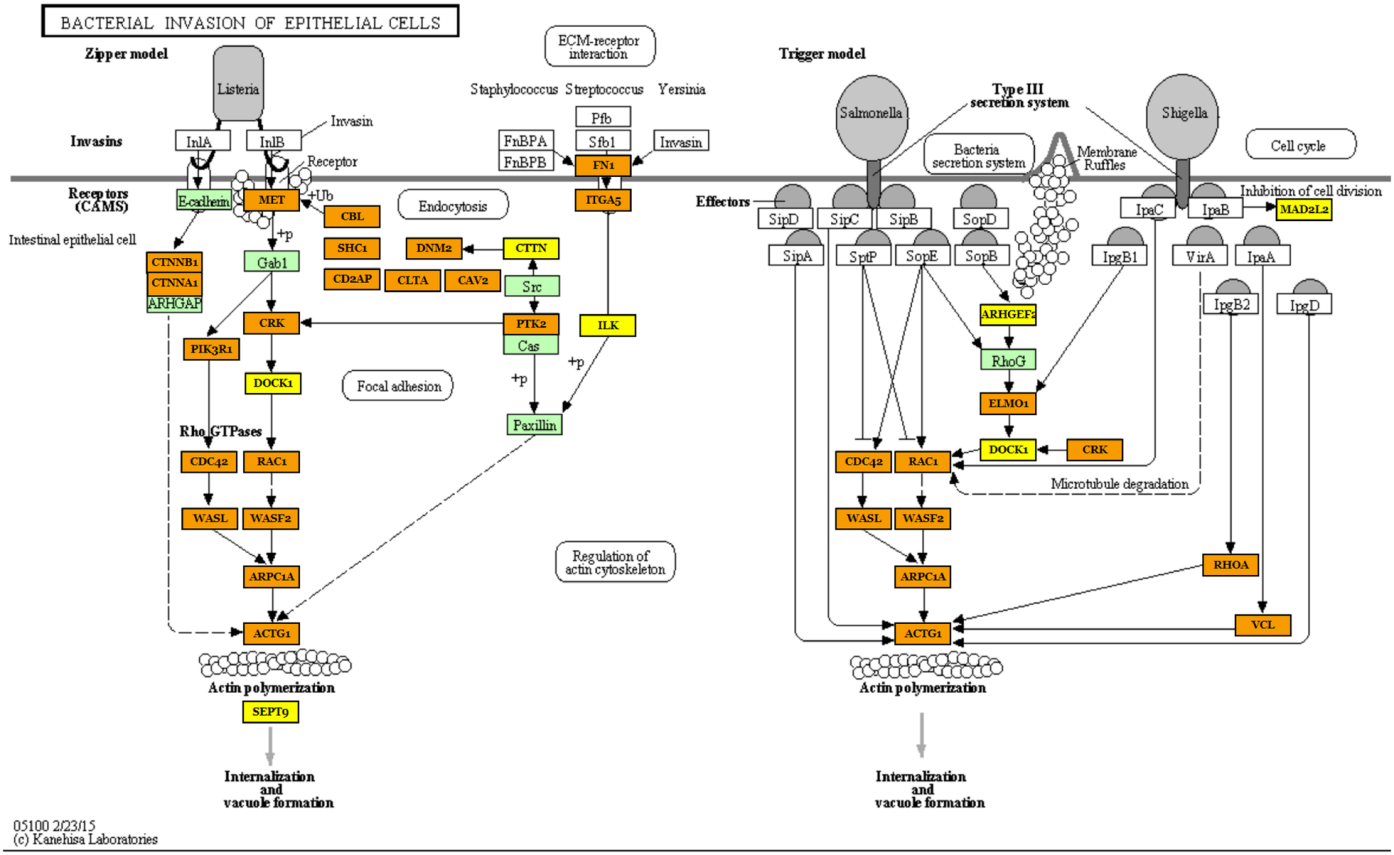
**Bacterial invasion of epithelial cells pathway (KEGG ID:hsa05100) in the CoPS vs. H comparison.** One of the most important pathways enriched by the differentially-expressed miRNAs. The green frame represents pathway-engaged genes. The target genes identified by the differential expression of miRNAs are marked yellow (included in one pathway) or orange (included in more than one pathway). Black arrows denote a molecular interaction or relation; dotted arrows represent an indirect link or unknown reaction. CDH1—E-Cadherin (Epithelial), CTNNB_1_—Catenin Beta 1, CTNNA_1_—Catenin Alpha 1, ARHGAP—GEM Interacting Protein, MET—MET Proto-Oncogene, Gab1—GRB2 Associated Binding Protein 1, CRK—CRK Proto-Oncogene, PIK3R1—Phosphoinositide-3-Kinase Regulatory Subunit 1, DOCK1—Dedicator Of Cytokinesis 1, CDC42—Cell Division Cycle 42, WASL—WASP Like Actin Nucleation Promoting Factor, RAC1—Rac Family Small GTPase 1, WASF2—WASP Family Member 2, ARPC1A—Actin Related Protein 2/3 Complex Subunit 1A, ACTG1—Actin Gamma 1, SEPT9—Septin 9, CBL—Cbl Proto-Oncogene, SHC1—SHC Adaptor Protein 1, CD2AP—CD2 Associated Protein, DNM2—Dynamin 2, CLTA—Clathrin Light Chain A, CAV2—Caveolin 2, CTTN—Cortactin, Src—SRC Proto-Oncogene, PTK2—Protein Tyrosine Kinase 2, Paxilin—PXN, FN1—Fibronectin 1, ITGA5—Integrin Subunit Alpha 5, ILK—Integrin Linked Kinase, CDC42—Cell Division Cycle 42, WASL—WASP Like Actin Nucleation Promoting Factor, RAC1—Rac Family Small GTPase 1, WASF2—WASP Family Member 2, ARHGEF2—Rho/Rac Guanine Nucleotide Exchange Factor 2, RhoG—Ras Homolog Family Member G, ELMO1—Engulfment And Cell Motility 1, DOCK1—Dedicator Of Cytokinesis 1, CRK—CRK Proto-Oncogene, MAD2L2—Mitotic Arrest Deficient 2 Like 2, RHOA—Ras Homolog Family Member A, ARPC1A—Actin Related Protein 2/3 Complex Subunit 1A, ACTG1—Actin Gamma 1, VCL—Vinculin.

Although the identified pathways appear to be associated with processes involved in infection, they are directed towards different types of pathogen in the analyzed comparisons. In tissues infected with CoPS, the most interesting enriched KEGG pathways were associated with *bacterial invasion of epithelial cells* (hsa05100) (process of bacteria entering into the epithelial cells), *endocytosis* (hsa04144), *focal adhesion* (hsa04510), *lysosome* (hsa04142), and *epithelial cell signaling in Helicobacter pylori infection* (hsa05120). Selected KEGG pathways and GO terms which were over-represented by DE miRNAs, together with examples of their target genes, are presented in Additional file 10, with full target gene names being given in Additional file 11. The first 20 GO terms with number of engaged genes and miRNAs are presented according to their *p*-value for CoPS vs. H (Figure 6) and CoNS vs. H (Figure 7). As an example, the *bacterial invasion of the epithelial cells pathway* is shown in detail in Figure 8. Staphylococci enter the cell using the zipper model, i.e. proteins expressed on the bacteria surfaces interact with cellular receptors, initiating signaling cascades, and resulting in the cellular membrane becoming tightly bound around the penetrating bacteria. The analysis also identified important infection-related GO terms related to *cell junction organization* (GO:0,034,330), *cell death* (GO:0,008,219), *immune system process* (GO:0,002,376) (any process related to immunity), and *signal transduction* (GO:0,007,165) (Additional file 10B; with full target gene names are given in Additional file 11).

**Figure 9:**
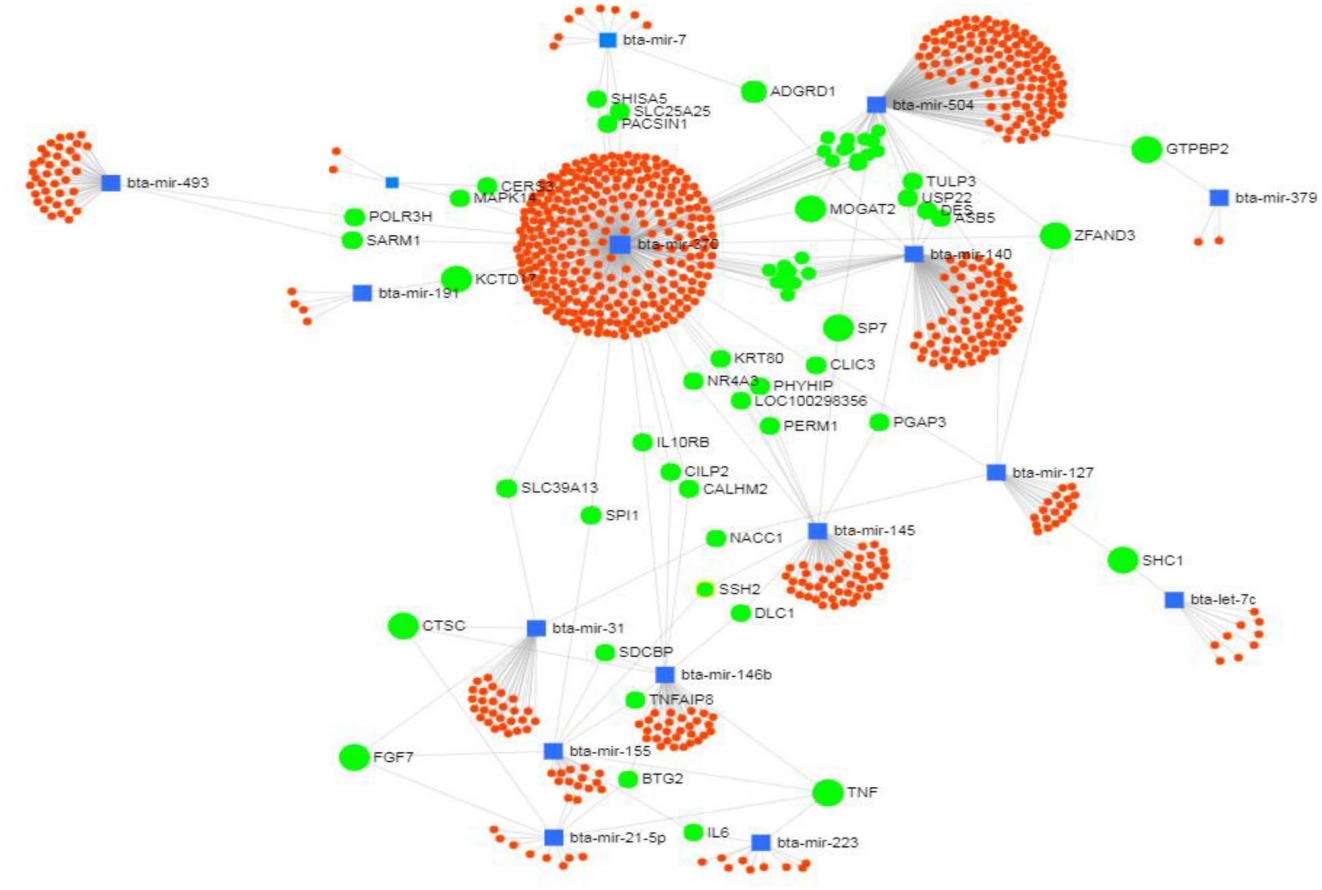
**Visualization of the identified differentially-expressed miRNAs and their target genes as an interaction network based on the common target genes (CoPS vs. H).** Blue squares represent differentially-expressed miRNAs, orange circles denote target genes, and green circles represent target genes common to 2 or more miRNAs. miRNet web application [67]. The abbreviations and full names of the target genes common to 2 or more miRNAs: POLR3H—RNA Polymerase III Subunit H, SARM1—Sterile Alpha And TIR Motif Containing 1, KCTD17—Potassium Channel Tetramerization Domain Containing 17, MAPK14—Mitogen-Activated Protein Kinase 14, CERS3—Ceramide Synthase 3, SHISA5—Shisa Family Member 5, SLC25A25—Solute Carrier Family 25 Member 25, PACSIN1—Protein Kinase C And Casein Kinase Substrate In Neurons 1, ADGRD1—Adhesion G Protein-Coupled Receptor D1, MOGAT2—Monoacylglycerol O-Acyltransferase 2, TULP3—TUB Like Protein 3, DES—Desmin, ASB5—Ankyrin Repeat And SOCS Box Containing 5, ZFAND3—Zinc Finger AN1-Type Containing 3, GTPBP2—GTP Binding Protein 2, SP7—Sp7 Transcription Factor, CLIC3—Chloride Intracellular Channel 3, KRT80—Keratin 80, NR4A3—Nuclear Receptor Subfamily 4 Group A Member 3, PHYHIP—Phytanoyl-CoA 2-Hydroxylase Interacting Protein, LOC100298356—Uncharacterized protein, PERM1—PPARGC1 And ESRR Induced Regulator, Muscle 1, PGAP3—Post-GPI Attachment To Proteins 3, SHC1—SHC Adaptor Protein 1, IL10RB—Interleukin 10 Receptor Subunit Beta, CLIP2—CAP-Gly Domain Containing Linker Protein 2, CALHM2—Calcium Homeostasis Modulator Family Member 2, SLC39A13—Solute Carrier Family 39 Member 13, SPI1—Spi-1 Proto-Oncogene, NACC1—Nucleus Accumbens Associated 1, SSH2—Slingshot Protein Phosphatase 2, DLC1—DLC1 Rho GTPase Activating Protein, CTSC—Cathepsin C, TNFAIP8—TNF Alpha Induced Protein 8, FGF7—Fibroblast Growth Factor 7, BTG2—BTG Anti-Proliferation Factor 2, TNF—Tumor Necrosis Factor, IL6—Interleukin 6.

An interaction network with the greatest number of target genes involved in the immune system is given as an example in Figure 9. This diagram shows how a large number of genes can be regulated by one or more different miRNAs. The in silico analysis found bta-miR-370 to be associated with the highest number of target genes. Some genes, such as zinc finger AN1-type containing 3 (*ZFAND3*), monoacylglycerol O-acyltransferase 2 (*MOGAT2*), cathepsin C (*CTSC*) and fibroblast growth factor 7 (*FGF7*) are influenced by 3 miRNAs, while Tumor Necrosis Factor (*TNF*) is regulated by as many as four.

### Differentially-expressed miRNAs engaged in infection-related pathways in the CoNS vs. H comparison

**Figure 10:**
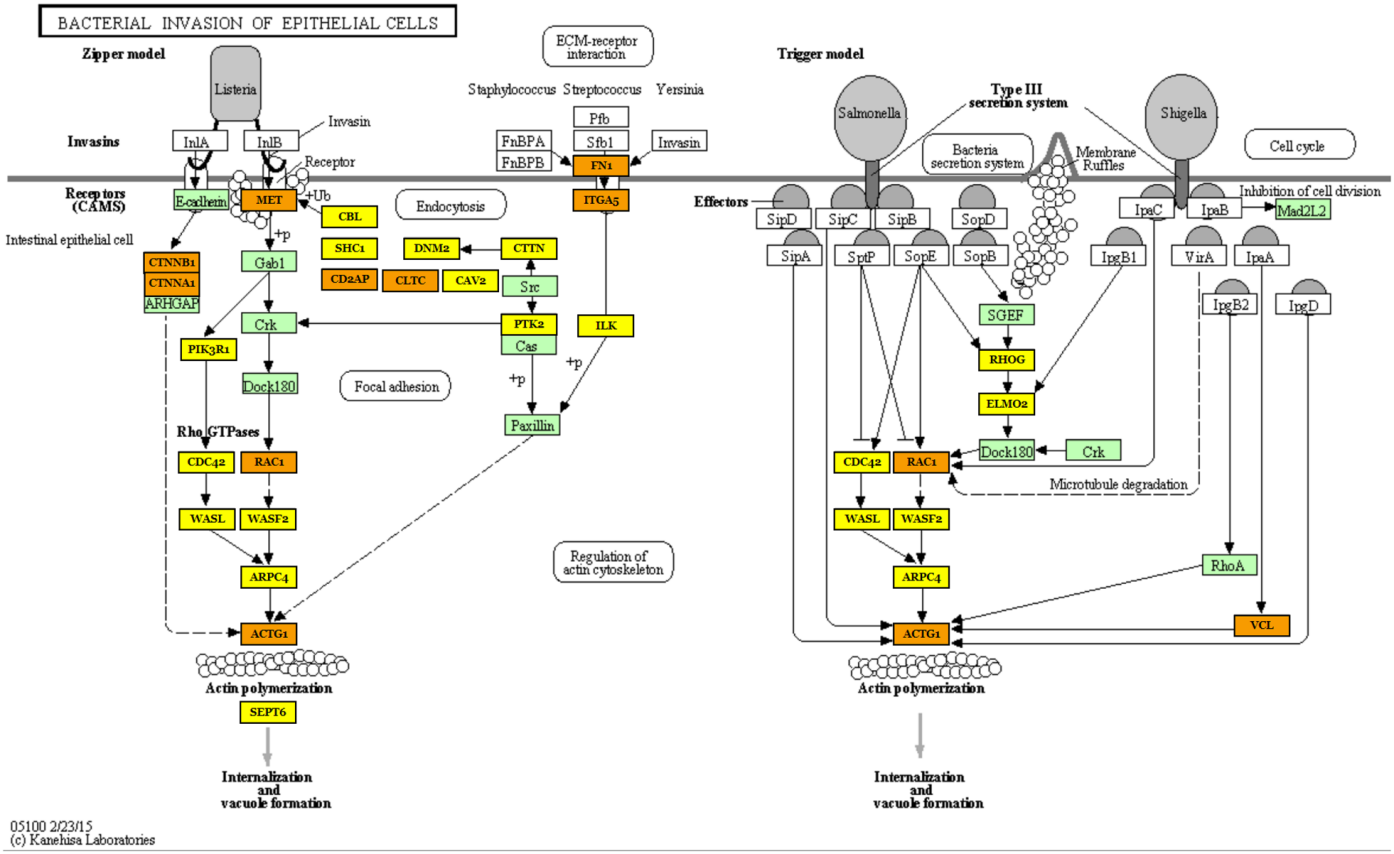
**Bacterial invasion of epithelial cells pathway (KEGG ID: hsa05100) in the CoNS vs. H comparison.** One of the most significantly regulated pathways by the differentially expressed miRNAs in the CoNS vs. H comparison. The green frame denotes pathway-engaged genes. The target genes identified by the differential expression of miRNAs are marked yellow (included in one pathway) or orange (included in more than one pathway). Black arrows denote molecular interactions or relations; dotted arrows represent indirect links or unknown reaction. CDH1—E-Cadherin (Epithelial), CTNNB_1_—Catenin Beta 1, CTNNA_1_—Catenin Alpha 1, ARHGAP—GEM Interacting Protein, MET—MET Proto-Oncogene, Gab1—GRB2 Associated Binding Protein 1, CRK—CRK Proto-Oncogene, PIK3R1—Phosphoinositide-3-Kinase Regulatory Subunit 1, DOCK180—Dedicator Of Cytokinesis 180, CDC42—Cell Division Cycle 42, WASL—WASP Like Actin Nucleation Promoting Factor, RAC1—Rac Family Small GTPase 1, WASF2—WASP Family Member 2, ARPC4—Actin Related Protein 2/3 Complex Subunit 4, ACTG1—Actin Gamma 1, SEPT6—Septin 6, CBL—Cbl Proto-Oncogene, SHC1—SHC Adaptor Protein 1, CD2AP—CD2 Associated Protein, DNM2—Dynamin 2, CLTC—Clathrin Light Chain C, CAV2—Caveolin 2, CTTN—Cortactin, Src—SRC Proto-Oncogene, PTK2—Protein Tyrosine Kinase 2, PXN—Paxilin, FN1—Fibronectin 1, ITGA5—Integrin Subunit Alpha 5, ILK—Integrin Linked Kinase, CDC42—Cell Division Cycle 42, WASL—WASP Like Actin Nucleation Promoting Factor, RAC1—Rac Family Small GTPase 1, WASF2—WASP Family Member 2, SGEF—Rho Guanine Nucleotide Exchange Factor 26, RhoG—Ras Homolog Family Member G, ELMO2—Engulfment And Cell Motility 2, DOCK180—Dedicator Of Cytokinesis 180, CRK—CRK Proto-Oncogene, MAD2L2—Mitotic Arrest Deficient 2 Like 2, RHOA—Ras Homolog Family Member A, ARPC4—Actin Related Protein 2/3 Complex Subunit 4, ACTG1—Actin Gamma 1, VCL—Vinculin.

**Figure 11:**
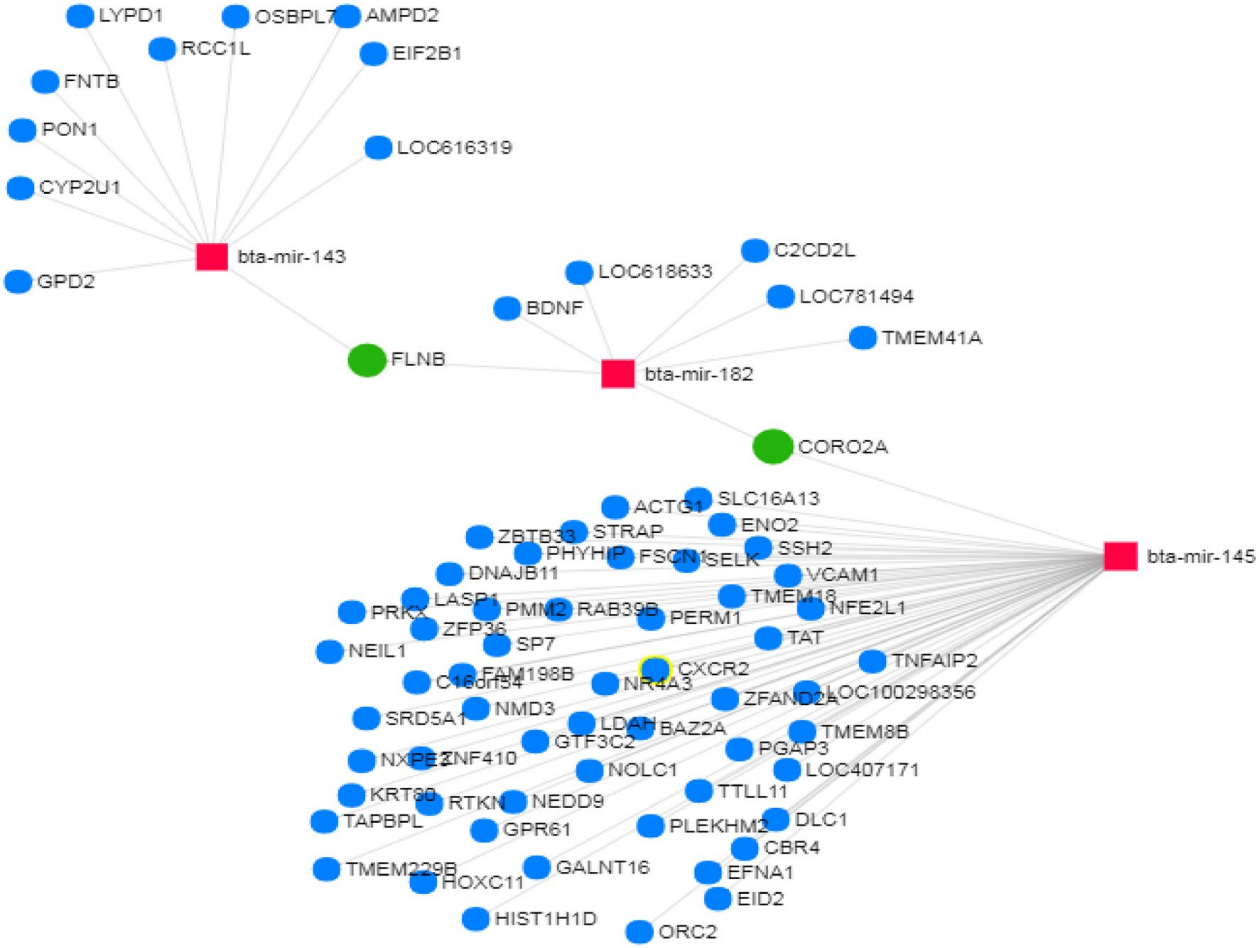
**Visualization of the identified differentially-expressed miRNAs and their target genes as the interaction network based on the common target genes (CoNS vs. H).** Blue squares represent differentially-expressed miRNAs, orange circles denote target genes, and green circles represent target genes common to two or more miRNAs. miRNet web application [67]. Red squares denote differentially expressed miRNAs, blue circles represent target genes, and green circles denote target genes common for two miRNAs. GPD2—Glycerol-3-Phosphate Dehydrogenase 2, CYP2U1- Cytochrome P450 Family 2 Subfamily U Member 1, PON1—Paraoxonase 1, FNTB—Farnesyltransferase, CAAX Box, Beta, LYPD1—LY6/PLAUR Domain Containing 1, RCC1L—RCC1 Like, OSBPL7—Oxysterol Binding Protein Like 7, AMPD2—Adenosine Monophosphate Deaminase 2, EIF2B1 -Eukaryotic Translation Initiation Factor 2B Subunit Alpha, LOC616319—Uncharacterized protein, FLNB—Filamin B, BDNF—Brain Derived Neurotrophic Factor, LOC618633—Uncharacterized protein, C2CD2L—C2CD2 Like, LOC781494—Uncharacterized protein, TMEM41A—Transmembrane Protein 41A, ORC2—Origin Recognition Complex Subunit 2, EID2—EP300 Interacting Inhibitor Of Differentiation 2, EFNA1—Ephrin A1, CBR4—Carbonyl Reductase 4, DLC1—DLC1 Rho GTPase Activating Protein, LOC470171—Uncharacterized protein, TMEM8B—Transmembrane Protein 8B, LOC100298356—Uncharacterized protein, TNFAIP2—TNF Alpha Induced Protein 2, ZFAND2A—Zinc Finger AN1-Type Containing 2A, PGAP3—Post-GPI Attachment To Proteins 3, TTLL11—Tubulin Tyrosine Ligase Like 11, PLEKHM2—Pleckstrin Homology And RUN Domain Containing M2, GALNT16—Polypeptide N-Acetylgalactosaminyltransferase 16, HIST1H1D—Histone Cluster 1 H1 Family Member D, HOXC11—Homeobox C11, GPR61—G Protein-Coupled Receptor 61, NEDD9—Neural Precursor Cell Expressed, Developmentally Down-Regulated 9, NOLC1—Nucleolar And Coiled-Body Phosphoprotein 1, BAZ2A—Bromodomain Adjacent To Zinc Finger Domain 2A, NFE2L1—Nuclear Factor, Erythroid 2 Like 1, TAT—Tyrosine Aminotransferase, CXCR2—C-X-C Motif Chemokine Receptor 2, NR4A3—Nuclear Receptor Subfamily 4 Group A Member 3, LDAH—Lipid Droplet Associated Hydrolase, RTKN—Rhotekin, TAPBPL—TAP Binding Protein Like, KRT80—Keratin 80, NXPE—Neurexophilin And PC-Esterase Domain Family Member 3, NMD3—NMD3 Ribosome Export Adaptor, SRD5A1—Steroid 5 Alpha-Reductase 1, FAM198B—Golgi Associated Kinase 1B, SP7—Sp7 Transcription Factor, PERM1—PPARGC1 And ESRR Induced Regulator, Muscle 1, TMEM18—Transmembrane Protein 18, VCAM1—Vascular Cell Adhesion Molecule 1, SSH2—Slingshot Protein Phosphatase 2, ENO2—Enolase 2, SLC16A13—Solute Carrier Family 16 Member 13, ACTG1—Actin Gamma 1, STRAP—Serine/Threonine Kinase Receptor Associated Protein, FSCN—Fascin Actin-Bundling Protein 1, SELK—Selenoprotein K, ZBTB33—Zinc Finger And BTB Domain Containing 33, DNAJB11—DnaJ Heat Shock Protein Family (Hsp40) Member B11, LASP1—LIM And SH3 Protein 1, PMM2—Phosphomannomutase 2, NEIL1—Nei Like DNA Glycosylase 1, PRKX—Protein Kinase X-Linked.

In tissues infected with CoNS, the most critical over-represented KEGG pathways included *adherens junction* (hsa04520), *bacterial invasion of epithelial cells* (hsa05100), *Shigellosis* (hsa05131), *Extracellular Matrix Receptor interaction* (hsa04512), and *focal adhesion* (hsa04510) (Additional file 10C; with full target gene names given in Additional 11). Infection-related GO categories, such as *extracellular matrix organization* (GO:0030198), *immune system process* (GO:0002376), *symbiosis, encompassing mutualism through parasitism* (GO:0044403), and *signal transduction* (GO:0007165) were also found to be over-represented (Additional file 10D; with full target gene names given in Additional file 11). Likewise, as for the CoPS vs. H comparison, *bacterial invasion of the epithelial cell pathway* is shown in detail in Figure 10. The lists of genes engaged in single or multiple pathways slightly differ between comparisons, with more genes involved in more than 2 pathways in the CoPS than the CoNS group. A clearer visualization of the interaction networks of DE miRNAs involving bta-miR-145, one of the miRNAs common to both comparisons, and their target genes are presented in Figure 11. As in Figure 9, it can be seen that a large number of genes appear to be regulated by single or multiple miRNAs; however, fewer miRNAs and target genes are involved in this CoNS network than in the example given from the CoPS vs. H comparison: only 3 miRNAs, viz. bta-miR-143, bta-miR-145 and bta-miR-182, are involved, with bta-miR-145 regulating the greatest number of genes. Only 2 genes are regulated by 2 miRNAs: filamin-B (*FLNB*) and coronin-2A (*CORO2A*).

### Differentially-expressed miRNAs engaged in infection-related pathways in both CoPS vs. H and CoNS vs. H comparisons

The next step focused on the pathways and categories identified in both comparisons (CoPS *vs*. H and CoNS vs. H) as represented by DE miRNA. The common KEGG pathway was the *bacterial invasion of epithelial cells*, while the common GO term was the *immune system process*. Information on each of the miRNA target genes described below was obtained from the available literature. The target genes of the DE miRNAs known to be involved in the *immune response* (i.e. 20 miRNAs with 49 target genes) or in the *bacterial invasion of epithelial cells* (20 miRNAs with 622 target genes) were selected for in silico analysis using mirPath v.3 DIANA Tools [28]. Some of these miRNAs were common to the identified KEGG pathways and GO categories associated with the *defense of epithelial cells during bacterial invasion* and *immune system processes*. The common miRNA genes for selected KEGG pathways and GO terms in the CoPS and CoNS cross-comparisons are listed in Tables 1 and 2, respectively. These miRNAs included *i.a*. miR-99b-5p, miR-155-5p, miR-191-5p, and miR-223-5p in the CoPS vs. H comparison, miR-182-5p in the CoNS vs. H comparison and miR-145-5p, miR-142-5p, and miR-31-5p in both comparisons (Additional files 3, 4 and 10; with full target gene names given in Additional file 11).

**Table 1:** **Common miRNAs genes in 2 selected KEGG pathways for CoPS vs. H and CoNS vs. H comparisons, over-represented by identified miRNAs.**

Pathway-comparison	No. of genes	Common miRNA genes
BI-CoPS BI-CoNS FA-CoPS FA-CoNS	7	miR-493-3p; miR-31-5p; miR-145-5p; miR-142-5p; miR-411-5p; miR-143-3p; miR-379-5p
BI-CoPS FA-CoPS	13	miR-199a-3p; miR-365a-3p; miR-23b-3p; let-7f-5p; miR-25-3p; miR-7-5p; miR-191-5p; miR-140-3p; miR-99b-5p; miR-146b-5p; miR-21-5p; miR-223-3p; miR-155-5p
BI-CoNS FA-CoNS	1	miR-182-5p
FA-CoPS FA-CoNS	2	miR-409-5p; miR-127-3p
FA-CoPS	1	miR-382-5p

CoPS—coagulase-positive staphylococci; CoNS—coagulase-negative staphylococci; H—healthy mammary gland group (without bacteria); KEGG—Kyoto Encyclopedia of Genes and Genomes

BI-CoPS—bacterial invasion of epithelial cells pathway (hsa05100) in CoPSvsH comparison;

BI-CoNS—bacterial invasion of epithelial cells pathway (hsa05100) in CoNSvsH comparison;

FA-CoPS—focal adhesion (hsa04510) in CoPSvsH comparison;

FA-CoPS—focal adhesion (hsa04510) in CoNSvsH comparison.

**Table 2:** **Common miRNAs genes in 2 selected GO term for CoPS vs. H and CoNS vs. H comparisons, over-represented by identified miRNAs.**

GO term-comparison	No. of genes	Common miRNA genes
ISP-CoPS ISP-CoNS ST-CoPS ST-CoNS	2	miR-493-3p; miR-145-5p
ISP-CoPS ISP-CoNS	4	miR-31-5p; miR-142-5p; miR-411-5p; miR-143-3p
ISP-CoPS ST-CoPS	4	miR-365a-3p; miR-23b-3p; miR-21-5p; miR-223-3p
ISP-CoPS	9	miR-199a-3p; let-7f-5p; miR-25-3p; miR-7-5p; miR-191-5p; miR-140-3p; miR-382-5p; miR-146b-5p; miR-155-5p
ISP-CoNS	1	miR-182-5p

CoPS—coagulase-positive staphylococci; CoNS—coagulase-negative staphylococci; H—healthy mammary gland group (without bacteria); GO—Gene Ontology

ISP-CoPS—immune system process (GO:0002376) in CoPSvsH comparison;

ISP-CoNS—immune system process (GO:0002376) in CoNSvsH comparison;

ST-CoPS—immune system process (GO:0002376) in CoPSvsH comparison;

ST-CoNS—signal transduction (GO:0007165) in CoPSvsH comparison.

The following genes are targets of the above-mentioned miRNAs, and are involved in the immune system: the CRK proto-oncogene (*CRK*) found in the *bacterial invasion of epithelial cells* KEGG pathway (CoPS vs. H); pellino E3 ubiquitin protein ligase 1 (*PELI1*) in the *immune system process* GO term (CoPS vs. H), dynamin 2 (*DNM2*) in the *bacterial invasion of epithelial cells* KEGG pathway (CoNS vs. H)*,* C-X-C chemokine receptor type 2 (*CXCR2*) in *immune system process* GO term (CoNS vs. H) (Additional file 10; with full target gene names given in Additional file 11). Moreover, based on the analysis of the interaction networks, bta-miR-370 and its target gene—interleukin 10 receptor subunit beta (*IL-10RB*) from the CoPS vs. H comparison (Figure 9), and bta-miR-145 with its target gene *CXCR2* (above-mentioned regarding *immune system process* GO term) from the CoNS vs. H comparison (Figure 11) were also selected for further analysis. In addition, the SHC-transforming protein 1, *alias* SHC Adaptor Protein 1 (*SHC1*) gene was found to be present in the *bacterial invasion of epithelial cells* KEGG pathway (Figures 8 and 10); it was also identified in the interaction network between the miRNAs and target genes identified in the CoPS vs. H comparison (Figure 9). This gene is known to be regulated by 2 miRNAs: bta-miR-127 and bta-let-7c. The protein products of these 3 target genes play important roles in the defense of the organism during inflammation and hence are discussed in detail herein.

## Discussion

Our study of the miRNAome of parenchyma samples from the udder quarters of dairy cows, based on a combination of NGS and bioinformatics analysis, indicates that twice as many DE miRNAs were identified in the CoPS vs. H than in the CoNS vs. H comparison. The numbers of miRNAs known to occur during clinical recurrent mastitis caused by *S. aureus* identified by Ju et al. [1] (*N* = 277) are similar to those reported in the present study (*N* = 256) (chronic subclinical mastitis); however, our present findings indicate a higher number of novel miRNAs (*N* = 260) than those presented by Ju et al. (*N* = 164) [1].

Among 48 common pathways identified for both comparisons, 2 of them, viz*. bacterial invasion of epithelial cells* (hsa05100), *focal adhesion* (hsa04510) were selected for further analysis. *TNF signaling pathway* (hsa04668) and *NF-kappa B signaling pathway* (hsa04064) directly involved in immunity, inflammation and modulation of immune responses, were identified only in CoPS vs. H comparison what is maybe be associated with the different mode of action and ability to survive in the host’s organism of these two groups of bacteria [4] as well as with the production capability of toxins that are also important virulence factor in infection pathogenesis [5]. This finding only partially confirms those from similar studies. Wang et al. [32] identified 33 significant KEGG pathways; of these, 7 were associated with immunity, including the *cytokine–cytokine receptor interaction*, *complement and coagulation cascades*, and *chemokine signaling pathway*. In addition, similarly to the present study, the *focal adhesion* pathway has also been found to be significantly altered during mastitis [32, 33]. Previous studies [1, 32] identified also less number of significant pathways than we did in our study (36 and 33 vs*.* 68 and 60 in CoPS vs*.* H and CoNS vs*.* H comparisons, respectively) (Additional files 6 and 7). However, our results confirmed those obtained by Ju et al. [1] since many of the pathways were the same in our study, including the *endocytosis*, *MAPK*, *canonical ErbB*, *TGF-beta*, or *wnt signaling pathways*. Similarly to us, among the GO biological processes, *immune system processes* and *signal transduction* were found by other authors to be enriched [32, 33]. More than fourfold higher numbers of various enriched GO terms were identified in Ju et al. [1] than in our study (249 vs. 56 in each comparisons) (Additional files 8 and 9). The elevated expression of the genes in the *bacterial invasion of epithelial cell* pathway indicates that the organism responded more strongly during CoPS invasion than CoNS invasion, since more genes in multiple pathways are expressed in the former than the latter. This finding is in agreement with the observation of Loof et al. [4] regarding the lower rate of persistency of CoNS which are more susceptible to immune system agents and are easier phagocytized than CoPS due to the lack of the coagulase production ability. Moreover, increasing in enterotoxin production by CoPS influences the severity of udder inflammation [5].

An analysis of the common miRNA genes in 2 selected KEGG pathways for CoPS vs*.* H and CoNS vs. H comparisons revealed 7 DE miRNAs which were identified in all subgroups in cross-comparisons e.g. *bacterial invasion of epithelial cell* × CoPS (BI-CoPS), *bacterial invasion of epithelial cell* × CoNS (BI-CoNS), *focal adhesion* × CoPs (FA-CoPS), and *focal adhesion* × CoNS (FA-CoNS) (Table 1). However, the highest number (13) of common DE miRNAs was found by comparing the BI-CoPS and BI-CoNS pathways. Considerably fewer common DE miRNAs were identified by cross-comparison of GO terms (*immune system process*—ISP and *signal transduction*—ST) and type of infection (Table 2). Only 2 miRNAs were common for all subgroups in cross-comparisons: miR-145-5p and miR-493-3p.

The fact that the miRNAs common to different pathways and GO terms were not always associated with immunity suggests that one miRNA may regulate the expression of many genes which, in turn, are involved in different pathways. In addition, it is possible that the metabolic pathways of infected cells can also be disturbed by the presence of the pathogen. Therefore we identified miRNAs influencing genes connected e.g. with the *lysosome* (hsa04142) and *endocytosis* (hsa04144) *pathways* (Additional file 10) which, in turn, are probably linked to each other, since substances digested in lysosomes are delivered to them using several processes including endocytosis [34]. Similarly, 18 DE miRNAs, with 438 target genes, are involved in the *cell death* GO term (GO:0008219) (Additional file 10) during CoPS infection. The GO descendants of *cell death* include *programmed necrotic cell death* (GO:0097300) and *apoptotic process* (GO:0006915).

Inter-individual variation in the groups was tested with the application of principal component analysis (PCA). The studied groups formed visible and distinct clusters, which confirmed the suitability of the groups for further differential expression analysis (Figures 1 and 2). One of the miRs whose expression was found to be upregulated in the KEGG pathways was miR-99b (Additional file 10A, CoPS vs*.* H; with full target gene names given in Additional file 11). Its expression has previously been found in adipose tissue, but not in mammary gland tissue [35]; however, unlike the present study, the samples were derived from non-lactating cows. miR-99b plays a crucial role in the pathogenesis of diseases caused by *Mycobacterium tuberculosis*, with strong expression being observed in infected dendritic cells and macrophages in cattle. Inhibition of miR-99b expression by synthetic antagomirs resulted in a significant decrease in bacterial growth in dendritic cells and increased expression of the pro-inflammatory cytokines IL-1, IL-6, IL-12 and Tumor Necrosis Factor alpha (TNFα) [36]. These factors affect various cells throughout the organism, not only leukocytes resulting in changes in cell and tissue morphogenesis, and various pathological processes. They can also have cytotoxic effects [37]. It is possible that miR-99b expression could be regulated not only by the host immune system but also by *M. tuberculosis* itself*.* A study of bovine responses to *S. aureus* found higher bta-miR-99b, bta-miR-2339, bta-miR-499 and bta-miR-23a expression in tissue exposed to pathogen compared to unexposed tissue [38]. However, its decreased expression in mammary gland tissue during acute mastitis was found [1]. miR-99b was also expressed in alveolar macrophages in healthy male Holstein–Friesian calves [39]. Similarly to Jin et al. [38], our findings indicate elevated expression of miR-99b during staphylococcal mastitis caused by CoPS but not CoNS, which may indicate that coagulase-positive staphylococci influence this particular miRNA, like *M. tuberculosis*.

Many of the miRNAs found to be downexpressed, such as miR-145, or overexpressed such as miR -31 and miR -155 in both CoPS and CoNS infected tissue in the present study (Additional files 3, 4, and 10A, B; with full target gene names given in Additional file 11), have also been previously identified in inflamed mammary gland parenchyma from Holstein–Friesian cows [40]. However, Li et al. [15] report a decreased level of miR-31 in mammary epithelial tissue infected by *S. aureus* compared to uninfected controls, while Ju et al. [1], found lower expression of all 3 miRNAs during acute *S. aureus*-induced mastitis. To date, miR-155 is one of the best-studied miRNAs, and is known to be involved in the maturation and functioning of B lymphocytes and the proper functioning of the immune response. Moreover, mice expressing the deficient miR-155 attachment site (bic/miR-155) have been found to demonstrate reduced humoral and cellular immune responses to infection [41]. This site is an exon of noncoding RNA, i.e. a primary miRNA precursor. Such mice were more susceptible to *Salmonella* spp. infection. They also demonstrated a reduced number of B lymphocytes, lower immunoglobulin levels and insufficient antigen presentation by dendritic cells [31, 42].

Another miRNA found to be overexpressed in the present study in both comparisons was miR-191 (Additional files 3, 4, and 10A, B; with full gene names given in Additional file 11). When overexpressed, this miRNA protects T lymphocytes from apoptosis induced by intracellular cytokine signals [43]. It is known to be expressed in the mammary gland tissue of non-lactating cows [35].

miR-223 was found to be elevated in CoPS-infected tissue but not in CoNS one (Additional files 3, 4, and 10A, B; with full gene names given in Additional file 11). It was found to be DE in bovine monocytes in response to stimulation by enterotoxin B from *S. aureus*, thus contributing to mastitis [44]; however, it has previously been found to be decreased during *S. aureus* infection [15]. Despite this discrepancy, miR-223 was found to have the highest expression of 48 DE genes in Mac-T cells, and it was proposed that this may alleviate the inflammatory pathways stimulated by *S. aureus*-derived LTA [45]. miR-223 is also believed to play a significant role in the unspecific immune response during myeloid differentiation, and in the functioning of granulocytes and their activation. Expression of miR-223 has been observed in bone marrow cells (e.g., hematopoietic progenitor cell antigen CD34 positive cells), but not T or B lymphocytes.

*CRK* is one of the 49 target genes of the miRNAs involved in the defense of mammary gland epithelial cells against the bacterial infections identified in the present study (Additional file 10A; with full gene names given in Additional file 11 and Figure 8—CoPS group). CRK is an oncoprotein that regulates transcription and cytoskeletal reorganization during cell growth, migration, and apoptosis by linking tyrosine kinases to small G proteins [46]. Its primary role is to act as a signaling molecule in regulating immune cell function. However, the precise nature of these processes remains unclear [47].

The DE miRs identified in the present study are also known to target *DNM2* (Additional file 10A, C; with full gene names given in Additional file 11 and Figure 8). This gene codes for dynamin 2, a ubiquitous protein involved in endocytosis and phagocytosis processes [48]. Dynamin GTPase 2 plays a key part in the late stage of these mechanisms by promoting the fission of the membrane surrounding the internalized material, thus facilitating the endocytosis of substances (Figure 8).

Another gene found in the CoPS vs. H interaction network (i.e. between DE miRNAs and target genes) (Figure 9), and which participates in the immune response, is *IL-10RB*, a target gene for miR-146b and miR-370, which were found to be upregulated in the present study in this comparison (Additional file 3). However, they were not DE in CoNS vs. H comparison. IL-10RB controls the host inflammatory response to microbial antigens; however, it mainly acts as a feedback inhibitor of the T cell response [49].

Although the involvement of miR-370 in the immune response remains poorly understood, its contribution to cancerogenesis and lipid metabolism have been studied in more detail [50]. Elevated miR-370 expression has been found to inhibit inflammation and oxidative stress [51], suggesting that it may support the defense against coagulase-positive staphylococci invasion; this is supported by the fact that miR-370 is one of 279 miRNAs found to be differentially expressed during bovine mastitis caused by *S. aureus* [52]. Therefore, the fact that the expression of miR-370 was elevated during mastitis caused by CoPS suggests that it may regulate some genes of the immune system. It might be, that CoPS can regulate also the mirR-370 gene in a similar way to miR-99b. It is important to note that miR-370 has a huge number of target genes, and a detailed analysis of their functions and interactions is needed to understand the epigenetic mechanisms occurring during staphylococcal mastitis.

In the present study, miR-142 was found to be upregulated in both comparisons (Additional files 3, 4, and 10; with full gene names given in Additional file 11). The miR-142 hairpin can give birth to 2 mature miRNAs encoded in the opposite strands: miR-142-3p and -142-5p. These miRNAs participate in the upregulation of leukocyte activity and reduction of inflammation. They are also known to affect the phenotype of T lymphocytes reacting during inflammation, as well as T cell differentiation, by targeting the transcripts of genes such as suppressor of cytokine signaling 1 (*SOCS1*) which are involved in *cytokine signaling pathway*. Both miRNAs are expressed in immune cells, and they maintain the homeostasis of dendritic cells. During an acute inflammatory reaction, neutrophils migrate immediately to the site of infection caused by pathogens (e.g., *S. aureus*). During this recruitment, miR-142 regulates the migration distance and velocity [53], although also found in the mammary glands of non-lactating cows [35].

In cow mammary gland parenchyma, miR-145 expression has been found to decrease during the inflammatory state [38]. One of miR-145 target genes is *CXCR2* (Additional file 10D; with full gene names given in Additional file 11, and Figure 11). To function correctly during infection, neutrophils require the activation of 2 chemokine receptors expressed on their surface: C-X-C motif chemokine receptor 1 (CXCR1) and CXCR2. Both CXCR1 and CXCR2 are activated by IL-8. Their action is interrelated, therefore a defect in only one receptor may still affect neutrophil activity. The recognition of chemokines by CXCR1 and CXCR2 results an increase in the expression of β2 integrin; this increase stimulates chemotaxis and reactive oxygen species production, as well as the phagocytosis of pathogens. Activation of CXCR1 and CXCR2 also protects neutrophils against spontaneous apoptosis [54]. CXCR2 may well be associated with the immune response during mastitis however, the direction of regulation depends on the type of infection, since it is a target gene of miR-145, which was found to be down-regulated in both comparisons.

The *SHC1* gene was identified in the *bacterial invasion of epithelial cells* pathway (Figure 8) and the *interaction network* in CoPS vs. H (Figure 9). The SHC1 protein is a signaling adapter that binds growth factor receptors to signaling pathways upon their activation. SHC1 is an essential element in the stimulation of the MAP/ERK kinase signaling cascade, which leads to suppression of the negative regulators of the cell cycle. It is also known to participate in many biological processes, including those connected with immunity, such as the *cytokine-mediated signaling pathway* (GO:0019221), *defense response to bacterium pathway* (GO:0042742), *interleukin-15-mediated signaling pathway* (GO:0035723), *interleukin-2-mediated signaling pathway* (GO:0038110), or *leukocyte migration* (GO:0050900) [55]. Although the *SHC1* gene is regulated by 2 miRNAs, only one of them, miR-127, was found to be up-regulated in both comparisons in the present study. In contrast, bta-let-7c was only found to be DE in the CoPS vs. H comparison. Unfortunately, little, if any, information currently exists on the role of miR-127 in bovine mastitis and only very limited data has been published regarding bta-let-7c during bovine mastitis [1, 35], thus, further study is needed on both miRNAs and their target genes.

Only one miRNA, bta-miR-106b-3p*, was found to be DE in the CoPS vs. CoPS and CoNS vs. CoNS comparison. It is a passenger strand not deposited in the miRBase so far. However, passenger strands are not always degraded and can have functional properties by targeting different mRNA populations [56]. miR-106b plays a role in many processes including those *associated with prion diseases* (hsa05020), *protein processing in endoplasmic reticulum* (hsa04141), or *TGF-beta signaling pathway* (hsa04350). Bta-miR-106 is responsible for triggering the expression of IL-10, which in turn triggers the expression of another 8 miRNAs; it also modulates the macrophage inflammatory response [57]. IL-10 is an anti-inflammatory cytokine produced later during inflammation that acts as a major suppressor of inflammatory activity [58]. However, IL-10 was not detected in milk during infection caused by *S. aureus* [59], although found at low levels in milk and blood serum during the subclinical form of inflammation caused by CoNS [60]. However, our present findings indicate that miR-106b level was elevated in the CoPS group compared to CoNS, suggesting that while it completely inhibits Il-10 expression in udder quarters infected by *S. aureus*, it only partly does so in those infected with CoNS; however, it is only a supposition. Moreover, more in-depth analyses of IL-10 expression are needed in all groups before any firm conclusions can be drawn.

The differences in miRNA profile observed between mammary gland tissues infected with CoPS and CoNS staphylococci can be attributed to the differences in their mode of action. In general, the staphylococcal mode of action during mastitis is based on its ability to release toxins into the intracellular space, as well as its potential to adhere to the host epithelial cell and potentially form a biofilm, and to evade the immune system by penetrating the host cell [4, 61]. Interestingly, none of the miRNAs found to be DE between CoPS vs. H and CoNS vs. H comparisons in our present study were related to the above-mentioned processes. Moreover, 66 of the same miRNA was DE during both types of infection. These all may suggest that both pathogen types (CoPS and CoNS) trigger genes participating in similar GO processes or KEGG pathways, but with different intensities: CoNS mastitis follows a milder course than *S. aureus* mastitis. It can be probably explained by the ability of CoPS to both coagulase and toxins production that both are important in the infection pathogenesis [4, 5].

Pattern recognition receptors (PRRs), such as TLRs and NOD-like receptors (NOD-nucleotide-binding oligomerization domain, NLRs), belong to innate response and are implicated in initial sensing bacterial components. They identified a pathogen-associated recognition pattern (PAMP) and triggered an innate response. Both CoPS, and CoNS have similar cell wall components, including LTA, which is recognized by TLRs and NLRs after bacterial invasion. Thus, at the beginning of the infection CoPS and CoNS may trigger similar mechanisms. However, during infection, modes of actions of these two groups of bacteria alter [62]. CoPS are known as more pathogenic strains, causing severe damage to host cells, producing and employing many virulence factors to ensure survival inside host tissue [63]. CoNS are considered opportunistic pathogens and less harmful to the host. However, this type of bacteria is also known for their virulence factors and ability to cause rather chronic than acute infection [64]. *S. aureus* genome-wide analysis revealed downregulation of virulence genes during colonization and their upregulation during infection propagation [65]. It may suggest a similar initial course of infection caused by both groups of pathogens by similar stimulation of PRRs, while the differences, found in our study, may be associated with implementing many other virulence factors, different for CoPS and CoNS at the later stage of infection since we used chronic model of mastitis.

Our results confirm that miRNAs play an essential role in immune system regulation in mammary gland secretory tissue during chronic mastitis. Increased expression of the identified miRNAs could inhibit the action of their targeted genes, such as those related to the immune response. It is possible that staphylococci, especially CoPS, can modulate the host immune response during inflammation. Based on the number of DE miRNAs involved in interaction networks, it may be assumed that CoPS trigger many more miRNAs genes than CoNS, and hence a higher number of target genes. CoNS bacteria, which are unable to produce coagulase to protect themselves against the immune system, are easier to fight and probably the host needs a much smaller arsenal of antimicrobial agents. Moreover, most of the CoNS strains do not produces toxins which are very important virulence factor and increase the severity of inflammation. Until now, there has been limited information on the roles of miRNAs, and further research should be carried out to elucidate their roles in the udder tissue and the course of infection with both types of staphylococci. However, some of the selected miRNAs such miR-99 or miR-181 may be used as new markers for subclinical mastitis diagnosing.

Our findings regarding the influence of the identified microRNAs on the etiology of mastitis serve as a further step towards understanding its molecular mechanisms and may allow more effective prevention and treatment, as well as functional studies on the role of microRNAs in the regulation of molecular pathways relevant to bacterial infection. They also provide many interesting molecular targets for genome editing-based functional studies on microRNA loci important for mastitis development.

## Supplementary Information

**Additional file 1. Characteristics of the identified miRNAs (CoPS vs. H). **Detailed table of miRNAs detected in all investigated samples with the use of the UEA sRNA Workbench software. “NA” denotes potentially novel miRNAs identified in this study.

**Additional file 2. Characteristics of the identified miRNAs (CoNS vs. H). **Detailed table of miRNAs detected in all investigated samples with the use of the UEA sRNA Workbench software. “NA” denotes potentially novel miRNAs identified in this study.

**Additional file 3. Results of the differential expression analysis using the DESeq2 algorithm (CoPS vs. H). **“baseMean” is the mean of normalized counts of all samples; “log2FoldChange” denotes the binary logarithm of the Fold Change parameter; “lfcSE” denotes log fold change Standard Error; “stat” stands for Wald statistics, that is, the log fold change divided by its standard error; “padj” denotes adjusted p-value; “NA” in the miRBAse ID column stands for potentially new miRNAs identified in this study.

**Additional file 4. The sequences and names of unique and common miRNA genes between comparisons.**

**Additional file 5. Results of the differential expression analysis using the DESeq2 algorithm (CoNS vs. H). **“baseMean” is the mean of normalized counts of all samples; “log2FoldChange” denotes the binary logarithm of the Fold Change parameter; “lfcSE” denotes log fold change Standard Error; “stat” stands for Wald statistics, that is, the log fold change divided by its standard error; “padj” denotes adjusted *p*-value; “NA” in the miRBAse ID column stands for potentially new miRNAs identified in this study.

**Additional file 6. List of KEGG pathways enriched by the detected differentially expressed miRNAs (p-value ≤ 0.05) (CoPS vs. H).**

**Additional file 7. List of KEGG terms enriched by the identified differentially expressed miRNAs (p-value ≤ 0.05) (CoNS vs. H).**

**Additional file 8. List of GO pathways enriched by the detected differentially expressed miRNAs (p-value ≤ 0.05) (CoPS vs. H).**

**Additional file 9. List of GO terms enriched by the identified differentially expressed miRNAs (p-value≤0.05) (CoNS vs. H).**

**Additional file 10. Identified differentially expressed miRNAs involved in KEGG pathways and GO categories. **Four tables containing selected KEGG pathway and GO categories with identified differentially expressed miRNAs and their exemplary target genes in the CoPS vs. H and CoNS vs. H comparisons.

**Additional file 11. Four tables containing the abbreviations and full names of the genes contained in the tables in Additional file 9 (KEGG pathways and GO categories).**

## Data Availability

The datasets supporting the conclusions of this article are included within the article and its additional files.

## Abbreviations

miRNA: MicroRNA
H: Non-infected tissue
CoPS: Coagulase-positive staphylococci
CoNS: Coagulase-negative staphylococci
KEGG: Kyoto Encyclopedia of Genes and Genomes
GO: Gene Ontology
TLR: Toll-like receptors
LPS: Lipopolysaccharide
nt: Nucleotide
TNF-α: Tumor necrosis factor alpha
IL-1β: Interleukin 1 beta
MyD88: Myleoid differentiation factor 88
NF-κB: Nuclear factor kappa-light-chain-enhancer of activated B cells
MAPK: Mitogen-activated protein kinases
LTA: Lipoteichoic acid
IL-8: Interleukin 8
GM-CSF: Granulocyte–macrophage colony-stimulating factor
NGS: Next generation sequencing
API: Analytical profile index
ZFAND3: Zinc finger AN1-type containing 3
MOGAT2: Monoacylglycerol *O*-acyltransferase 2
CTSC: Cathepsin C
FGF7: Fibroblast growth factor 7
IL-6: Interleukin 6
TNFAIP8: TNF alpha induced protein 8
IL10RB: Interleukin 10 receptor subunit beta
GTPBP2: GTP binding protein 2
MAPK14: Mitogen-activated protein kinase 14
SP7: Sp7 transcription factor
CRK: CRK proto-oncogene
PELI1: Pellino E3 ubiquitin protein ligase 1
DNM 2: Dynamin 2
CXCRC1: C-X-C chemokine receptor type 1
CXCRC2: C-X-C chemokine receptor type 2
IL-12: Interleukin 12
IL-27: Interleukin 27
TGF-β: Transforming growth factor β
MFSD6: Major facilitator superfamily domain containing 6
DDIT4L: DNA damage-inducible transcript 4 like
CTPS1: Cytidine 5-prime triphosphate synthetase
DNMT3A: DNA cytosine-5- methyltransferase 3A
FOXO1: Forkhead box protein O1
Ago: Argonaute proteins
SOCS1: Suppressor of cytokine signaling 1
MHC: Major histocompatibility complex
DE: Differential expression
