# Supplementary material for: MicroRNA expression profile in bovine mammary gland parenchyma infected by coagulase-positive or coagulase-negative staphylococci
Source: Vet Res. 2021 Mar 6;52:41. doi: 10.1186/s13567-021-00912-2 (PMC7937231; doi:10.1186/s13567-021-00912-2)
Supplement: Supplementary file 10 — Additional file 10. Identified differentially expressed miRNAs involved in KEGG pathways and GO categories. Four tables containing selected KEGG pathway and GO categories with identified differentially expressed miRNAs and their exemplary target genes in the CoPS vs. H and CoNS vs. H comparisons. [file 13567_2021_912_MOESM10_ESM.docx]

**Additional file 10A Selected KEGG pathways over-represented by identified miRNAs, their exemplary target genes, CoPS vs. H comparison.**

| **KEGG pathway** | **miRNAs involved in a pathway** | **No of target genes** | **Exemplary target genes** | ***P*-value** |
| --- | --- | --- | --- | --- |
| bacterial invasion of epithelial cells (hsa05100) | hsa-miR-7-5p, **hsa-miR-145-5p**, hsa-miR-21-5p, hsa-miR-191-5p, hsa-let-7f-5p, hsa-miR-199a-3p, hsa-miR-155-5p, hsa-miR-411-5p, hsa-miR-23b-3p, hsa-miR-140-3p, **hsa-miR-99b-5p,** hsa-miR-25-3p, hsa-miR-365a-3p, hsa-miR-143-3p, hsa-miR-146b-5p, hsa-miR-31-5p, **hsa-miR-142-5p**, hsa-miR-223-3p, hsa-miR-493-3p, hsa-miR-379-5p | 49 | *ARPC5, ACTB, ARPC5L, MET, WASF1, ITGB1, WASL, CBL, DNM2, CRKL, SEPT8, CRK, SHC1, PIK3CB, CAV1, SEPT11, ACTG1, ITGA5, ELMO2, CLTC, VCL, RHOA* | 6.7764e-05 |
| endocytosis (hsa04144) | hsa-miR-155-5p, hsa-miR-7-5p, **hsa-miR-145-5p**, hsa-miR-21-5p, hsa-miR-199a-3p, hsa-miR-143-3p, hsa-let-7f-5p, hsa-miR-146b-5p, hsa-miR-140-3p, hsa-miR-23b-3p, hsa-miR-25-3p, hsa-miR-382-5p, hsa-miR-31-5p, **hsa-miR-142-5p**, **hsa-miR-99b-5p**, hsa-miR-365a-3p, hsa-miR-411-5p, hsa-miR-191-5p, hsa-miR-493-3p, hsa-miR-223-3p, hsa-miR-127-3p, hsa-miR-409-5p, hsa-miR-379-5p; | 118 | *RNF41, VPS4A, RAB4A, ARAP2, MET, PDGFRA, ARFGEF1, HSPA1A, TGFBR1, CHMP7, VPS45, ADRBK1, CBL, SMAD2, DNM2, FGFR3, SH3KBP1, ARF3, WWP1, DAB2, PIP5K1C, PRKCI, CYTH2, CAV1, AP2B1, SMURF2, IL2RB, NEDD4L, HLA-E;* | 0.0001 |
| focal adhesion (hsa04510) | hsa-miR-155-5p, hsa-miR-25-3p, hsa-miR-140-3p, hsa-miR-23b-3p, hsa-miR-191-5p, hsa-miR-199a-3p, **hsa-miR-145-5p**, hsa-miR-21-5p, hsa-miR-31-5p, hsa-miR-7-5p, hsa-let-7f-5p, hsa-miR-146b-5p, hsa-miR-127-3p, hsa-miR-143-3p, hsa-miR-365a-3p, **hsa-miR-142-5p**, hsa-miR-493-3p, hsa-miR-379-5p, hsa-miR-382-5p, **hsa-miR-99b-5p**, hsa-miR-411-5p, hsa-miR-223-3p, hsa-miR-409-5p; | 122 | *ACTB, ITGB1, MAPK14, ACTG1, ITGA5, DIAPH1, RAC1, PFN1, NFKB1, WASF1, ITGB1, WASL, BTRC, NFKBIB, ACTB, NFKB1, VCL, MAPK9, MAPK8, RAC1, PFN1, ARPC1B, CD44, ARPC5, PFN1, ELMO2, ARPC1B, DIAPH1, MAPK1, CD44;*  *ACTG1, MAPK1, ITGA5;* | 0.0002 |
| lysosome (hsa04142) | hsa-miR-23b-3p, hsa-miR-146b-5p, hsa-miR-199a-3p, hsa-miR-7-5p, hsa-miR-191-5p, hsa-miR-143-3p, **hsa-miR-142-5p**, hsa-let-7f-5p, hsa-miR-155-5p, hsa-miR-493-3p, hsa-miR-140-3p, hsa-miR-379-5p, hsa-miR-21-5p, **hsa-miR-145-5p**, hsa-miR-25-3p, hsa-miR-365a-3p, hsa-miR-31-5p, hsa-miR-382-5p, hsa-miR-127-3p; | 67 | *HGSNAT, NAGA, AP4B1, ABCA2, M6PR, AP3S1, GNS, AP1G2, HEXB, SORT1, IDS, SGSH, SLC17A5, ARSB, CTSD, CD164, CLTC, CTSC, GM2A, PSAP*; | 0.0295 |
| epithelial cell signaling in Helicobacter pylori infection (hsa05120) | hsa-miR-23b-3p, hsa-miR-143-3p, hsa-miR-146b-5p, hsa-miR-140-3p, hsa-miR-155-5p, hsa-miR-7-5p, hsa-miR-365a-3p, **hsa-miR-145-5p**, **hsa-miR-99b-5p**, hsa-miR-31-5p, hsa-miR-191-5p, hsa-let-7f-5p, **hsa-miR-142-5p**, hsa-miR-199a-3p, hsa-miR-21-5p, hsa-miR-493-3p, hsa-miR-25-3p, hsa-miR-379-5p, hsa-miR-382-5p; | 40 | *HBEGF, ADAM10, NFKB1, ATP6V0E1, MET, CXCL8, MAPK14, ATP6V1F, PAK1, CHUK, ATP6V1D, ATP6V1E1, EGFR, TJP1, ATP6V1G1, ATP6V1H, ADAM17, ATP6V1B2, MAP3K14*; | 0.0383 |

CoPS – coagulase-positive staphylococci; H – healthy mammary gland group (without bacteria); KEGG - Kyoto Encyclopedia of Genes and Genomes. Full names of genes in the 10^th^ supplement.

Highlighted miRNA are described in the main text in detail.

**Additional file 10B** **Selected GO terms over-represented by identified miRNAs, their exemplary target genes, CoPS vs. H comparison.**

| **GO term** | **miRNAs involved in a pathway** | **No of target genes** | **Exemplary target genes** | ***P*-value** |
| --- | --- | --- | --- | --- |
| cell junction organization (GO:0034330) | hsa-miR-143-3p, **hsa-miR-145-5p**, hsa-miR-25-3p, hsa-miR-199a-3p, hsa-miR-21-5p, hsa-miR-23b-3p, hsa-miR-23b-3p, hsa-miR-155-5p, hsa-miR-7-5p, hsa-let-7f-5p | 79 | *ACTB, TLN2, PRKCA, PTPRK, ACTN2, FERMT2, CDH2, CDH13, ITGB1, PLEC, FLNC, CTNND1, LAMA5, APC, CDH6, PIP5K1C, PRKCI, DLG1, PVR, TAOK2, ACTG1KRT14, TESK2, SNAI2, SMAD3, ITGB4;* | <1e-325 |
| cell death (GO:0008219) | hsa-miR-143-3p, **hsa-miR-145-5p**, hsa-miR-493-3p, hsa-miR-25-3p, hsa-miR-146b-5p, hsa-miR-199a-3p, hsa-miR-21-5p, hsa-miR-140-3p, hsa-**miR-142-5p**, hsa-miR-23b-3p, hsa-miR-23b-3p, hsa-miR-155-5p, hsa-miR-31-5p, hsa-miR-7-5p, hsa-miR-365a-3p, hsa-miR-382-5p, hsa-miR-191-5p, hsa-let-7f-5p | 438 | *RTN4, VAPB, SLK, ESPL1, PFN1, TNFRSF11B, STK17A, GLRX2, ZMAT3, DFNA5, BICD2, CDIP1, ALS2, DUSP22, CLU, SIGMAR1, UBE2Z, CSNK2A2, FZD5, E2F1, PDCD4, TGFBR1, ADAM10, NFKB1, FEM1B, YWHAH*; | <1e-325 |
| immune system process (GO:0002376) | hsa-miR-223-3p, hsa-miR-143-3p, hsa-miR-411-5p, **hsa-miR-145-5p**, hsa-miR-493-3p, hsa-miR-25-3p, hsa-miR-146b-5p, hsa-miR-199a-3p, hsa-miR-21-5p, hsa-miR-140-3p, hsa-**miR-142-5p**, hsa-miR-23b-3p, hsa-miR-23b-3p, hsa-miR-155-5p, hsa-miR-31-5p, hsa-miR-7-5p, hsa-miR-365a-3p, hsa-miR-382-5p, hsa-miR-191-5p, hsa-let-7f-5p | 622 | *ARPC5, B2M, PELI1, TRIB3, BRK1, IRS2, ADAM9, ACTB, DUSP4, PHLPP2, RAB4A, PRLR, FOS, ERAP1, TCF3, WIPF2, GSK3B, CXADR, MOV10, SOX4, HBEGF, PRKCA, TNFSF4, IL1RAP, CLU, TRIM11, POLR3D, PDE4B, PDGFRA, GLG1, RBCK1, TAB1, FZD5*; | <1e-325 |
| signal transduction (GO:0007165) | hsa-miR-223-3p, **hsa-miR-145-5p**, hsa-miR-493-3p, hsa-miR-21-5p, hsa-miR-23b-3p, hsa-miR-23b-3p, hsa-miR-365a-3p | 850 | *AHI1, FZD7, CCNT2, RTN4, DDR1, TAOK3, ESR1, E2F7, PELI1, RAB2B, PLXNA2, BRK1, IRS2, GABBR1, FHL2, TNFRSF11B;* | 3.7900e-05 |

CoPS – coagulase-positive staphylococci; H – healthy mammary gland group (without bacteria); GO - Gene Ontology

Full names of genes in the 10^th^ supplement.

Highlighted miRNA are described in the main text in detail.

**Additional file 10C Selected KEGG pathways over-represented by identified miRNAs, their exemplary target genes, CoNS vs. H comparison.**

| **KEGG pathway** | **miRNAs engaged in a pathway** | **No of target genes** | **Exemplary target genes** | ***P*-value** |
| --- | --- | --- | --- | --- |
| adherens junction (hsa04520) | **hsa-miR-145-5p**  hsa-miR-182-5p  hsa-miR-493-3p  hsa-miR-31-5p  hsa-miR-143-3p  hsa-miR-379-5p  **hsa-miR-142-5p**  hsa-miR-127-3p | 13  22  4  6  5  1  5  1 | *ACTB, SMAD2, ACTG1, SNAI2, IGF1R, EGFR, SNAI1;*  *MET, WASF1, WASL, PVRL2, IQGAP1, PTPN1, IGF1R, VCL, WASF3, TJP1;*  *IQGAP1, SMAD3, CDH1, FGFR1;*  *ACTB, MET, VCL, CTNNA1 , RAC1, MAP3K7;*  *CSNK2A2, LMO7, CTNNA1, EP300, MAPK1;*  *CREBBP;*  *ACTG1, FER, EP300, YES1, MAPK1;*  *LMO7;* | 5.7429e-09 |
| bacterial invasion of epithelial cells (hsa05100) | hsa-miR-182-5p  hsa-miR-31-5p  hsa-miR-143-3p  **hsa-miR-145-5p**  hsa-miR-411-5p  **hsa-miR-142-5p**  hsa-miR-493-3p  hsa-miR-379-5p | 22  6  5  13  3  2  2  1 | *MET, WASF1, ITGB1, WASL, CBL, DNM2, SHC1, PIK3CB, ARPC3, CLTC;*  *ACTB, MET, CLTC , VCL, CTNNA1, RAC1;*  *ARPC5, ELMO2, ARPC1B, CTNNA1, SEPT3;*  *ACTB, ITGB1, CAV1, ACTG1, ITGA5, CLTC, CAV2;*  *PIK3CB, SEPT11, FN1;*  *ACTG1, CD2AP;*  *ARPC1B, CDH1;*  *ITGA5* | 1.8473e-06 |
| Shigellosis (hsa05131) | **hsa-miR-145-5p**  hsa-miR-182-5p  hsa-miR-31-5p  hsa-miR-493-3p  hsa-miR-143-3p  **hsa-miR-142-5p**  hsa-miR-379-5p | 7  21  6  3  7  2  1 | *ACTB, ITGB1, MAPK14, ACTG1, ITGA5, DIAPH1, RAC1;*  *PFN1, NFKB1, WASF1, ITGB1, WASL, BTRC,NFKBIB;*  *ACTB, NFKB1, VCL, MAPK9, MAPK8, RAC1;*  *PFN1, ARPC1B, CD44;*  *ARPC5, PFN1, ELMO2, ARPC1B, DIAPH1, MAPK1, CD44;*  *ACTG1, MAPK1;*  *ITGA5;* | 1.1823e-05 |
| ECM-receptor interaction (hsa04512) | hsa-miR-493-3p  hsa-miR-143-3p  hsa-miR-31-5p  **hsa-miR-145-5p**  hsa-miR-182-5p  hsa-miR-127-3p  hsa-miR-411-5p  **hsa-miR-142-5p**  hsa-miR-379-5p | 5  5  3  15  8  2  3  1  3 | *THBS2, ITGAV, COL5A1, LAMC2, CD44;*  *THBS1, COL5A1, COL1A1, LAMC1, CD44;*  *SDC1, COL1A1, LAMC1;*  *ITGB1, THBS1, ITGA5, ITGA3, COL3A1;*  *ITGB1, ITGB8, THBS1, LAMA1, COL3A1,*  *DAG1, LAMC1, SDC4;*  *THBS1, ITGA3;*  *THBS2, COL4A2, FN1;*  *ITGAV;*  *ITGA5, COL6A1, COL5A1;* | 3.1217e-05 |
| focal adhesion (hsa04510) | **hsa-miR-142-5p**  hsa-miR-182-5p  hsa-miR-143-3p  hsa-miR-127-3p  **hsa-miR-145-5p**  hsa-miR-31-5p  hsa-miR-411-5p  hsa-miR-379-5p  hsa-miR-409-5p  hsa-miR-493-3p | 7  34  9  3  34  10  7  3  1  6 | *ACTG1, ITGAV, CCND1, SOS1, PTEN, MAPK1, XIAP;*  *GSK3B, MET, ITGB1, ITGB8, SHC1, HBS1, PIK3CB, PAK2, CCND2, ROCK2;*  *THBS1, BCL2, DIAPH1, COL5A1, COL1A1, LAMC1, PTEN, MAPK1, TLN1;*  *THBS1, ITGA3, FLNA;*  *ACTB, GSK3B, PRKCA, CAPN2, ITGB1, THBS1, CAV1, ACTG1, ITGA5;*  *ACTB, MET, PPP1CC, VCL, MAPK9, CCND1, MAPK8, COL1A1, RAC1, LAMC1;*  *PDGFRA, PIK3CB, THBS2, CCND1, COL4A2, FN1, BIRC3;*  *ITGA5, COL6A1, COL5A1;*  *VEGFA;*  *THBS2, ITGAV, JUN, COL5A1, PDGFC, LAMC2;* | 0.0038 |

CoNS – coagulase-negative staphylococci; H – healthy mammary gland group (without bacteria); KEGG - Kyoto Encyclopedia of Genes and Genomes. Full names of genes in the 10t^h^ supplement.

Highlighted miRNA are described in the main text in detail.

**Additional file 10D** **Selected GO terms over-represented by identified miRNAs, their exemplary target genes, CoNS vs. H comparison.**

| **GO term** | **miRNAs engaged in a pathway** | **No of target genes** | **Exemplary Target genes** | ***P*-value** |
| --- | --- | --- | --- | --- |
| extracellular matrix organization (GO:0030198) | **hsa-miR-145-5p**  hsa-miR-493-3p  hsa-miR-379-5p | 39  14  6 | *DDR1, MMP3, PRKCA, SPARC, ITGB1, PLEC, COL12A1, THBS1, ITGA5, ITGA3;*  *SPARC, COL12A1, VCAM1, CTSD, HAS2, TIMP2, ITGAV, NID2;*  *ITGA5, COL6A1, CASP3, COL5A1, SERPINE1;* | <1e-325 |
| immune system process (GO:0002376) | **hsa-miR-145-5p**  hsa-miR-493-3p  hsa-miR-411-5p  hsa-miR-143-3p  **hsa-miR-142-5p**  hsa-miR-182-5p  hsa-miR-31-5p | 78  27  9  60  56  170  43 | *IRS2, ACTB, TCF3, GSK3B, PRKCA, ITGB1, CD59, SLC7A6, INPPL1, IFI30;*  *HBEGF, SMAD3, TUBB, VCAM1, CTSD, UBC, CANX, TNRC6B;*  *PDGFRA, IFI30, PIK3CB, CEBPB;*  *ARPC5, WIPF2, GLG1, ADAM10, BNIP3, SLC7A6, CALM3, CALM1, THBS1, MAPK7, LGR4;*  *B2M, PELI1, PHLPP2, PDE4B, IRF4, BNIP3, IRF9, CD80, CALM1, ADAR;*  *TRIB3, PHLPP2, PRLR, GSK3B, SOX4, CEBPG, PRKCQ, TAB1, ADAM10, NFKBI;*  *ACTB, IL1RAP, NFKB1, IL1R1, CD59, CALM1, HIST1H2BK, AGO3, PIK3R4, KIF2A;* | <1e-325 |
| symbiosis, encompassing mutualism through parasitism (GO:0044403) | **hsa-miR-145-5p**  hsa-miR-493-3p  hsa-miR-379-5p  hsa-miR-411-5p  hsa-miR-143-3p  **hsa-miR-142-5p**  hsa-miR-182-5p  hsa-miR-31-5p | 35  14  9  4  43  30  117  33 | *HIPK2, STAT3, ITGB1, HMGA1, PCSK5, CAV1, AP2B1, ANPEP, ITGA5, PSMD3;*  *RPL3, ANPEP, RPL37, SRCAP, SMAD3, VCAM1, UBC, UBB, RRAGA;*  *ANPEP, ITGA5, POLR2A, KDM4A, RPS16, EIF4G1, HSPA8, DDX39B, CREBBP;*  *ACKR3, PDGFRA, HNRNPA1, POLR2A;*  *HIPK2, CRTC3, PACS1, POLR2C, BNIP3, TAF10, NUP153, USP6NL, ZMYND11;*  *VAPB, B2M, TNPO1, TRIM23, BNIP3, CD80, ANKRD17, CCNH, TCEB1, MAVS;*  *ACKR3, HIPK2, CRTC3, CDK7, STAT3, TNPO1, RPS23, UBP1, ITGB1, NUP85;*  *TBC1D20, CRTC3 , STAT3, YWHAE, NUP210, SRCAP, UBA52, SLC25A5, ARF1;* | <1e-325 |
| signal transduction (GO:0007165) | **hsa-miR-145-5p**  hsa-miR-493-3p | 159  53 | *FZD7, DDR1, ESR1, IRS2, ACTB, HIPK2, GSK3B, PRKCA, ARL6IP5, SPARC, STAT3, G3BP1, PLXNB2, ITGB1, AKR1B10;*  *FHL2, TNFRSF11B, PTK7, HBEGF, SPARC, PLXNB2, HNF4A, LPHN2, B9D1,*  *IQGAP1;* | 0.000167697 |

CoNS – coagulase-negative staphylococci; H – healthy mammary gland group (without bacteria); GO - Gene Ontology

Full names of genes in the 10^th^ supplement.

Highlighted miRNA are described in the main text in detail.
